# Evaluation of the Safety and Potential Benefits of Beetroot-Based Dietary Supplements According to Their Elemental Composition

**DOI:** 10.1007/s12011-023-03902-x

**Published:** 2023-10-07

**Authors:** Joanna Brzezińska-Rojek, Małgorzata Rutkowska, Justyna Ośko, Piotr Konieczka, Magdalena Prokopowicz, Małgorzata Grembecka

**Affiliations:** 1 Department of Bromatology, Faculty of Pharmacy, Medical University of Gdańsk, Gen. J. Hallera Avenue 107, 80-416, Gdansk, Poland; 2 Department of Analytical Chemistry, Faculty of Chemistry, Gdańsk University of Technology, Gabriela Narutowicza 11/12 Street, 80-233, Gdansk, Poland; 3 Department of Physical Chemistry, Faculty of Pharmacy, Medical University of Gdańsk, Gen. J. Hallera Avenue 107, 80-416, Gdansk, Poland

**Keywords:** Beetroot, Dietary supplements, Mineral composition, Toxic elements, Food safety, Cadmium

## Abstract

The study aimed to assess the health value and safety associated with the consumption of the chosen 37 beetroot-based dietary supplements (DSs). An optimized and validated analytical procedure, using a method called microwave plasma atomic emission spectrometry (MP-AES), was developed to determine the profiles of 19 elements (Na, K, Fe, Ca, Pt, Zn, Cd, Cu, V, Co, Ni, Pb, Mo, Mg, Al, Mn, Sr, Cr, Ba) in the DSs. The products were assessed for compliance with the recommended daily doses for the chosen elements, and any deviations were identified. Results showed that powders constituted a richer source of elements than capsules and tablets. The exception was iron-enriched products, which provided the highest dose of Fe (3.75 to 25 mg/daily dose). Safety assessment was evaluated in 3 steps, including (1) the determination of levels of Al, Ba, Cd, and Pb; (2) comparison of their content with the permissible contamination limits; and (3) comparison of the weekly or monthly intake of Al and Cd with the provisional tolerable weekly (PTWI) or monthly (PTMI) intake, respectively. The content of Ba was evaluated because of the oral reference dose (RfD). Five products were significantly contaminated with Cd (4–134% of PTMI Cd), two with Al (11–12%), and three with Ba (1.085–1.331 µg/d.d.). Lead was not detected above the LOQ (0.035 mg/kg). Factor analysis was employed to differentiate between the pharmaceutical forms (capsules, tablets, and powders) and determine the origin of the powder contained in the capsules. These results highlight the importance of implementing more stringent control measures and regulatory changes in the DSs market to ensure consumer safety.

**Supplementary Information:**

The online version contains supplementary material available at 10.1007/s12011-023-03902-x.

## Introduction

Plant preserves are willingly used to produce dietary supplements (DSs). Beetroot preserves such as lyophilizate, dried beetroot, dried juice, concentrates, and extracts are examples of this. This vegetable (*Beta vulgaris* L.) is a rich source of bioactive substances and minerals [1, 2], with its mineral compound content ranging from 0.5 to 2.5% [3]. It is also a valuable source of macroelements such as sodium (Na), potassium (K), magnesium (Mg), and calcium (Ca), as well as microelements such as manganese (Mn), iron (Fe), zinc (Zn), and copper (Cu) [4–6]. Mineral compounds play a part in metabolic and physiological processes in the human body [7]. Electrolytes such as K, Na, Ca, and Mg play a crucial role in maintaining osmotic and electrolyte balance [8]. Sodium and K regulate the transmission of electrical impulses and the acid–base balance in human cells [9]. Calcium is a component of the human skeletal system and teeth; it is necessary for the blood clotting process and, along with Na, participates in muscle contraction [9, 10]. Magnesium is engaged in skeleton building, thermoregulation, and blood pressure regulation [11, 12]. Magnesium, Mn, Cu, Zn, and Fe are involved in synthesizing and activating enzymes [11, 13–16]. Manganese participates in the physiology of the sexual, nervous, skeletal, and circulatory systems, as well as in the metabolism of cholesterol, carbohydrates, and red blood cells [13, 17]. Zinc is crucial for the proper functioning of the circulatory and immune systems [18]. Copper affects the synthesis of proteins and neurotransmitters, gene expression, the absorption of Fe, the deactivation of free radicals, and the processes of generating energy in cells [14]. Iron is an element with redox properties and, as a component of hemoglobin, is involved in the delivery of oxygen to cells. In addition, it is necessary for the proper functioning of the nervous system and immune system and is also involved in detoxification processes in the liver [16, 19].

As a root vegetable, beetroot tends to accumulate heavy metals, such as cadmium (Cd), mercury (Hg), and lead (Pb) [20, 21]. Moreover, some studies have found a high Zn and Mn content in beetroots grown near industrialized or mining areas [21, 22]. Toxic metals interrupt plant metabolism by changing the electrical potential of the cell membrane and the pH value of the cytoplasm. Consequently, the mineral and bioactive composition of the vegetable might be decreased [3]. It was found that beetroot, next to carrots, provides the highest amounts of heavy metals among vegetables [21, 23]. The level of contaminants in foodstuffs results from food type, growing and meteorological conditions (such as soil type, water, and atmospheric deposition), agricultural and cultivation procedures (such as using fertilizers containing Cd or other metals), and anthropogenic contamination of the environment [24]. Cadmium, Pb, and Zn have been released into the environment as a result of natural and anthropogenic processes such as volcanic eruptions, mining, and the extraction of metals from ores [21].

Prolonged exposure to Cd might result in the accumulation of this element in the kidneys, leading to nephropathy. Furthermore, Cd intake leads to disorders of Ca metabolism, resulting in the formation of kidney stones or disorders of bone turnover such as osteomalacia and osteoporosis [25]. IARC classified Cd and its compounds as carcinogenic to humans (Group 1). Cadmium alters DNA repair and tumor-suppressor proteins, leading to chromosomal damage and instability. Transduction process disturbances lead to the deregulation of cell growth. Chromosomal damage, genomic instability, epigenetic alterations, and direct binding to DNA appear to be of minor importance [25, 26].

Chronic Pb consumption leads to anemia, neurological disorders (headaches, irritability, depression, seizures, muscle weakness, ataxia, tremors, and hearing impairment), as well as gastroenterological disorders and renal failure [27]. Moreover, it is associated with an increased risk of hypertension, coronary heart disease, stroke, and probably an increased risk of cancerogenesis (Group 2a). Organic compounds are classified as non-carcinogenic (Group 3). Lead causes male fertility disorders in the form of a reduced sperm count and an increased number of damaged sperm [27].

To ensure the safety of consumers and reduce food contamination as far as possible, especially because of increased dietary supplement (DS) consumption, the European Commission has set maximum permissible levels (ML) of contaminants in foodstuffs, such as 1 mg/kg of Cd and 3 mg/kg of Pb in DSs [28, 29]. Member States of the European Union are obligated to enforce food law and monitor and verify that the relevant requirements of food law are fulfilled by food producers, including DS manufacturers. Contaminated products in quantities exceeding ML must not be placed on the market. Furthermore, the World Health Organization (WHO) established a provisional tolerable monthly intake (PTMI) of Cd amounting to 25 µg/kg b.w./month [30]. Due to the high toxicity of Pb, the provisional tolerable weekly intake (PTWI) value was withdrawn because it was impossible to establish a safe dose of this element [31]. The PTWI and PTMI indicators are determined for food contaminants that can accumulate in the human organism and are expressed per kg of body weight. They refer to the appropriate weekly and monthly consumption from all sources that does not have a negative impact on health.

DSs are concentrated forms of food intended to supplement the diet. A small amount of such products might provide a notable dose of components, which is why consumers find them convenient [32]. Although they are governed by food regulations, the Chief Sanitary Inspector (GIS) in Poland only controls a limited number of products after they are put on the market. The GIS must be notified before products are released, but there is no obligation to provide analyses of such products prior to their entry into the market [32]. This effortless procedure results in many notifications of new products being launched, and GIS is receiving more notifications than they can inspect. Thus, there is a justified concern that some products on the market are of insufficient quality and may even be health-threatening [33–36]. Since January 2023, the Draft Law on Amendments to the Law on Food and Nutrition Safety in Poland [37, 38] has been processed, and it is motivated by the necessity to adjust to the constantly shifting marketplace for these products. These include, among other things, improving the transparency of advertisements in this category to highlight their lack of medicinal properties. The advertising message is the main source of information about DSs for most consumers, and the marketing techniques used affect the consumer’s awareness, thereby indirectly affecting the decision to purchase a specific food category and, thus, the quality of consumed products. In addition, a time regime is proposed for supplementing the documentation by the manufacturer at the request of the Chief Sanitary Inspectorate. What’s more, it is suggested to allow the mark confirming the quality and safety of the product based on the analysis of its composition, provided that an opinion from the appropriate scientific unit is submitted.

Although there is considerable research on beetroot [39–41], there is still a lack of studies on the beetroot-based DS elemental composition and contaminants. However, some pioneering studies on such areas as prescription food for special medical purposes (FSMPs) and modified milk products (MMPs) for newborns and infants available in Polish pharmacies were conducted [42–44]. They did not show any real health hazards to newborns and infants associated with the investigated samples [42, 45, 46]. Nevertheless, the composition of FSMPs and MMPs formulas ready to use differed from the manufacturer’s declaration [46]. Furthermore, research highlights the need for verification of the Ni content in these products [42]. Safety and risk assessment research on metal contamination as well as the elemental composition of beetroot-based DSs are essential from a nutritional and toxicological perspective.

This study aimed to evaluate the safety and quality of 37 DSs based on their elemental content. The obtained results were compared with dietary recommendations, permissible levels of contamination, and toxicity parameters measuring exposure to harmful substances, such as PTWI, PTMI, and the oral reference dose (RfD). Moreover, the content of the chosen elements in the analyzed DSs was compared with the results for vegetables obtained in our previous study, which referred to the elemental composition of DSs and beetroot [43]. The reason for undertaking this type of research is the fact that many of these products are available for sale in pharmacies, but nobody can guarantee their safety because they are not routinely toxicologically analyzed. Significant amounts of DSs are available for sale online, including untrustworthy sources, and, thus, may pose a risk to consumers. Particularly athletes may take large amounts of beetroot-based products to increase exercise tolerance over extended periods of time, potentially putting themselves at risk of heavy metal poisoning. Obtained results can be beneficial for other researchers, DS manufacturers, and institutions monitoring the food market, such as the Chief Sanitary Inspectorate.

## Materials and Methods

### Sample Preparation

Thirty-seven supplements consisting of beetroot were obtained from online or in-person shops in the Polish market. The detailed characteristics are shown in Table S1. Products in tablet form were marked with *T*, capsules with *C*, and powder with *P*. Manufacturers declared Fe content in seven products (marked with * in Table S1). The analysis included products that were available in the form of capsules, tablets, or powders, contained beetroot preserves (i.e., dried juice, powdered root, dried extracts, or lyophilizate) as a main ingredient (>50%), and were accessible to Polish consumers in person or online. Every product was analyzed in triplicate with threefold measurements. The products were purchased twice (in November 2020 and in October 2021) to obtain a representative group of products in terms of availability during this period. Products were homogenized directly before analysis using ceramic tools. Determination was carried out according to a previously published procedure [43].

### Reagents and Standards

The following concentrations of standard solutions were acquired from Sigma-Aldrich (Germany): 1000 ± 2 mg/L of K, Ba, Ca, Cd, Co, Cu, Zn, Ni, Pb, Pt, V, and Mo; 1006 ± 4 mg/L of Mg; 1001 ± 2 mg/L of Fe; and 998 ± 5 mg/L of Al. Standards of Na (10 000 mg/L) and Sr (1005 ± 5 mg/L in 4% HNO_3_) were obtained from MSSpectrum (Poland), while a chromium (Cr) standard at a concentration of 1003 ± 3 µg/mL and an Mn standard at a concentration of 1000 ± 6 µg/mL were purchased from CPI International (USA). Nitric acid (65–70% purity) was obtained from Alfa Aestar (Germany).

Agilent’s 4210 MP-AES high-sensitivity atomic emissions were used, along with the Millipore Milli-Q Water Purification System (USA) and Anton Paar Multiwave Go microwave mineralizer.

### Determination Procedure

The determination of the elements in the tested samples, which were previously mineralized, was carried out using atomic emission spectrometry with microwave plasma atomization (via the 4210 MP-AES supplied by Agilent) at specific wavelengths for each element (Table 1).

**Table 1:** Validation parameters of the procedure for the determination of selected elements in samples of beetroot-based food supplements (Standard deviation of the slope (S_b_), standard deviation of the intercept (S_a_), residual standard deviation (S_*x*,*y*_))

Analyte	Wavelength (nm)	LOD (mg/kg)	LOQ (mg/kg)	Linearity								
				Calibration range [mg/kg]								
				Minimum concentration	Maximum concentration	No. of measurement points	Number of repetitions	Calibration curve	*R*^2^	S_a_	S_b_	S_x,y_
Na	568.263	1.1	3.3	10	200	5	4	*y* = 34.46*x −* 34.1	0.9998	6.1	0.12	16
K	766.491	0.16	0.48	2.5	20	4	4	*y* = 48347*x −* 16941	0.9997	1780	269	2907
Fe	371.993	0.33	1.0	1.0	100	8	4	*y* = 5510*x −* 1049	0.9997	329	23	1109
Ca	430.253	2.0	6.0	10	250	6	4	*y* = 898.5*x* + 1376	0.9995	249	3.1	157
Pt	265.945	0.075	0.23	0.40	4.0	4	4	*y* = 3628.1*x* + 498.9	0.9994	8.3	6.7	211
Zn	213.857	0.19	0.58	0.58	10	9	4	*y* = 12014*x* + 96.1	0.9995	4.9	287	187
Cd	228.802	0.022	0.066	0.066	20	8	4	*y* = 23459*x −* 524.9	0.9998	1652	9.3	285
Mg	279.553	0.40	1.2	1.2	40	6	4	*y* = 152325*x* + 37340	0.9996	2166	341	1047
Pb	405.781	0.012	0.035	0.050	5.0	6	4	*y* = 2775.9*x −* 77.29	0.9999	0.98	4.7	463
Cu	327.395	0.026	0.077	0.30	20	6	4	*y* = 44555*x −* 1626	0.9999	1057	153	652
Co	345.351	0.012	0.035	0.050	1.0	5	4	*y* = 13331*x −* 2.4	0.9999	754	1.8	471
Ni	361.939	0.0070	0.021	0.10	20	7	4	*y* = 5637*x −* 337.9	0.9999	8.2	105	398
Mo	386.410	0.0060	0.018	0.018	20	9	4	*y* = 15860*x* + 29	0.9995	1074	13	257
Al	396.152	0.088	0.26	1.0	100	8	4	*y* = 20008*x* + 632	0.9998	836	85	147
Mn	403.076	0.0064	0.019	0.019	1	5	4	*y* = 28990*x* + 44	0.9999	1003	10	211
Sr	421.552	0.0045	0.013	0.013	40	6	4	*y* = 29277*x* + 58	1.0000	1458	23	130
Cr	425.433	0.0027	0.0082	0.01	10	8	4	*y* = 29402*x* + 29	0.9999	1051	13	245
Ba	493.408	0.21	0.63	0.63	3.0	4	4	*y* = 20318*x* + 5708	0.9962	1620	549	680
V	437.923	0.0057	0.017	0.017	20	9	4	*y* = 7795*x* + 42	0.9997	109	17	133

### Method Validation

The validation of the mercury/MA-3000 method was performed by the linearity, the limit of determination (LOD), and the limit of quantification (LOQ), precision, and accuracy. The LOD and LOQ of the applied method were calculated using formulas proposed by Huber [44]:

1 $$\mathrm{LOD}= \frac{3.3\;{\mathrm{SD}}_{\mathrm{a}}}{\mathrm{b}}$$

SD_a_: standard deviation of the intercept for the calibration curve
b: slope of the calibration curve

When calculating the numerical LOQ, the dependence described by Huber [44] was used:

2 $$\mathrm{LOQ}=3\bullet \mathrm{LOD}$$

The determination coefficients (*R*^2^) were in the range of 0.9862–1.0000. The average recovery for the selected elements (Na, K, Ca, Mg, Fe, Zn, Cu, Ag, Co, Al, Ni, Mo, Mn, Sr, Cr, Ba, Pb, Cd, V) was in the range of 80–120%, which are acceptable values in such analyses (Table 1). Precision was calculated as the coefficient of variation for all the results obtained in all analyzed samples. Values were obtained at an acceptable level and did not exceed 10%. Recovery for calibration curves (R_cc_) was calculated based on the signal obtained for standards (S_expected_) and the signal calculated from the calibration equation (S_calculated_) using the following formula:

3 $${\mathrm{R}}_{\mathrm{cc}}=\frac{\left|{\mathrm{S}}_{\mathrm{expected}}- {\mathrm{S}}_{\mathrm{calculated}}\right|}{{\mathrm{S}}_{\mathrm{expected}}}$$

### Calculations

#### Content Calculations

The content of particular elements was determined as µg/g of each product and then calculated into µg per daily dose (d.d.). The particular results for each product are presented in Tables S2 and S3 as the mean content in a product ± expanded uncertainty (U) of measurement at a 95% confidence level obtained for the threefold measurement.

#### Intake Assessment

By using DSs following the manufacturer’s recommendation (Table S1), the consumer is provided with a specific amount of the analyzed elements, which has been expressed as the estimated daily intake (EDI). The estimated weekly intake (EWI) and estimated monthly intake (EMI) were obtained by multiplying the EDI by 7 and 30, respectively.

#### Realization of Dietary Recommendations and Safety Assessment

The adequate intake (AI) and recommended dietary allowance (RDA) values for adult males (19–75 years old) were adopted for the estimation of health value according to recommendations for the Polish population [47]. The calculated EDI of the analyzed DSs was compared with RDA values or with AI where RDA was not given (Na, K, and Mn). Moreover, for Fe-enriched products, the compliance of the Fe content with the manufacturers’ declarations was assessed (Table S1) according to the recommendations of the European Commission [48, 49].

The safety of the analyzed DSs was estimated according to European Commission regulations No. 1881/2006 and No. 629/2008 [28, 29], while human exposure was assessed by comparing the EDI index to the PTWI, PTMI, or RfD values. According to the *United States Pharmacopoeia* (*USP* 43-NF 38), manufacturers of supplements are encouraged to estimate the content of elemental contaminants (As, Cd, Hg, Pb) and estimate the health risk based on PTWI, which is recommended by the United Nations Food and Agriculture Organization (FAO) and WHO [50].

#### Statistical Analysis

All data were checked for normal distribution [51]. The data obtained were characterized by a non-normal distribution, so non-parametric tests such as the Kruskal-Wallis test and Spearman rank correlation were performed. The results obtained were subjected to factor analysis using Statistica 13.3 (TIBCO Software Inc., Palo Alto, CA, USA). The analyses confirmed the authenticity of the natural raw material (beetroot) in the products and provided a distribution in terms of the pharmaceutical form of the products (DSs containing beetroot), the level of Fe enrichment, and the form of the main plant material used (beetroot in the DS). The comparative data on the natural raw beetroot material were sourced from Brzezińska-Rojek et al.’s [43] study and were used to further develop the chemometric analyses conducted.

## Results and Discussion

The content of 19 elements (Na, K, Fe, Ca, Pt, Zn, Cd, Cu, V, Co, Ni, Pb, Mo, Mg, Al, Mn, Sr, Cr, and Ba) was determined. The average results for the three groups of DSs (tablets, capsules, and powders) are presented in Table 2. The results are expressed as µg/d.d. of the DSs based on the manufacturers’ recommendations. The concentrations of Pt (LOQ = 0.23 mg/kg), Ni (LOQ = 0.021 mg/kg), and Pb (LOQ = 0.035 mg/kg) were under the LOQ in all samples. Moreover, in tablets and capsules, a concentration of V above the LOQ was not detected. In all the powder samples, the Cd content was above the LOQ.

**Table 2:** Elemental composition of DSs in tablets, capsules, or powders and realization of dietary recommendations for adult males from 19 to 75 years old by a daily portion of the analyzed dietary supplements

	Analyzed element	Dietary Recommendations (mg/day)	*n*^1^	(µg/daily dose)							The realization of dietary recommendations (%)
				Mean	SD	Min	Median	Max	Q1	Q3	
Tablets	Na	1500^a^	10	2303	759	1260	2650	3198	1540	2848	0.2^a^
	K	3500^a^	10	6963	5470	22	7117	15313	2074	11613	0.199^a^
	Mg	420^b^	10	1855	1400	336	1642	4350	710	2841	0.46^b^
	Ca	1000^b^	10	44,067	95,399	60	1193	309,165	558	44,209	4.4^b^
	Fe	10^b^	10	2660	3973	19	104	11,438	51	3722	27^b^
	Mn	2.3^a^	9	25	11	8.7	25	41	17	32	1.096^a^
	Zn	11^b^	6	5.9	4.5	0.78	5.7	10.8	2.2	9.8	0.053^b^
	Cu	0.9^c^	1	3.2	NC	NC	NC	NC	NC	NC	0.066^a^
	Al	NR	10	14	9.1	2.6	11	28	6.7	19	NC
	Ba	NR	10	2.2	1.2	0.39	2.4	3.9	1.1	3.0	NC
	Cd	NR	2	34	43	3	34	64	19	49	NC
	Co	NR	1	1.97	NC	NC	NC	NC	NC	NC	NC
	Cr	NR	7	1.3	1.7	0.19	0.53	4.5	0.29	1.6	NC
	Mo	NR	10	6.1	7.001	0.11	4.3	21	0.57	7.6	NC
	Sr	NR	9	10	14	1.9	6.2	48	3.3	7.6	NC
Capsules	Na	1500^a^	14	5265	5193	194	3752	15,760	1474	5887	0.4^a^
	K	3500^a^	14	11,313	9442	640	9621	40,338	8022	11,760	0.32^a^
	Mg	420^b^	14	1327	1308	151	1052	5396	509	1684	0.33^b^
	Ca	1000^b^	14	917	1269	86	462	4890	270	831	0.092^b^
	Fe	10^b^	14	2104	6588	9.1	46	24,678	32	100	21^b^
	Mn	2.3^a^	14	22	27	0.29	15	102	3.5	27	0.95^a^
	Zn	11^b^	9	14	14	4.3	7.9	47	7.5	15	0.13^b^
	Cu	0.9^b^	4	3.6	2.5	1.4	3.3	6.5	1.6	5.4	0.074^b^
	Al	NR	14	51	162	0.70	6.5	614	3.1	8.5	NC
	Ba	NR	14	35	99	0.38	2.1	374	1.4	6.9	NC
	Cd	NR	3	28	43	2.3	3.8	78	3.1	41	NC
	Co	NR	1	3.2	NC	NC	NC	NC	NC	NC	NC
	Cr	NR	3	1.2	0.88	0.19	1.3	1.9	0.76	1.6	NC
	Mo	NR	14	5.1	11	0.046	0.31	35	0.20	1.1	NC
	Sr	NR	14	3.5	5.6	0.68	1.1	17	0.91	2.3	NC
Powders	Na	1500^a^	13	61,267	69,097	784	32,523	203,874	19,710	49,120	4.1^a^
	K	3500^a^	13	14,3503	110,892	4977	140,650	403,524	54,576	17,1630	4.1^a^
	Mg	420^b^	13	16,743	11,474	418	17,869	37,005	8124	23,391	4.2^b^
	Ca	1000^b^	13	17,292	15,028	83	17,846	48,870	4518	26,772	1.7^b^
	Fe	10^b^	13	2048	3491	20	741	12,786	452	1105	20^b^
	Mn	2.3^a^	13	246	279	1.9	149	1058	77	341	11^a^
	Zn	11^b^	12	186	125	4.5	179	398	91	250	1.7^b^
	Cu	0.9^b^	11	40	30	11	32	94	18	55	0.81^b^
	Sr	NR	13	102	89	0.65	80	320	64	113	NC
	Al	NR	13	471	817	1.6	128	2420	28	411	NC
	Ba	NR	13	517	473	0.96	545	1331	129	830	NC
	Cr	NR	4	7.3	8.4	0.39	5.025	19	1.3	11	NC
	Co	NR	3	17	17	2.8	13	35	7.99	24	NC
	Mo	NR	13	5.6	6.02	0.39	2.9	21	2.5	8.7	NC
	V	NR	2	3.0	2.5	1.2	2.97	4.7	2.1	3.8	NC

*SD*, standard deviation; *Min*, minimum; *Max*, maximum; *n*^1^, number of samples with the determined content of analyzed element above LOQ; ^a^AI for man, ^b^RDA for man; *NR*, lack of dietary recommendation; *NC*, not calculated due to lack of data, Cd in powders < LOQ (0.066 mg/kg), V in capsules and tablets < LOQ (0.017 mg/kg)

### Content of the Analyzed Elements

The tablets, capsules, and powders differed in terms of their elemental content. The variability of elemental contents in individual groups might be related to the different origins of the supplements, the composition of the beetroot preserves used in production, and the presence of auxiliary substances. The products came from different countries (Table S1) and were purchased from various stores. The formulations contained different auxiliary substances, such as anti-caking agents, acidity regulators, and sweeteners, which might also be sources of elements. The daily doses differed in all of the analyzed groups due to the characteristics of the formulations; powders administered the highest amounts, while tablets and capsules administered the lowest. The greatest amounts of elements were delivered from powders because their portions were much larger by weight (3–15 g/day) than the daily dose of tablets or capsules (0.35–4.4 g/day).

There is a lack of literature that allows us to compare data on DSs produced from *Beta vulgaris* L. The writers did, however, publish one article on the topic, and some of the findings were compared [43].

#### Macrominerals in DSs

Potassium was the most abundant macromineral in capsules (11 mg/d.d.) and powders (144 mg/d.d.) while tablets delivered the highest dose of Ca (44 mg/d.d.) (Table 2). The tablet product T9 was distinguished by a significantly higher Ca content than the other DSs (126 mg/d.d.). The manufacturer stated that one of the excipients in this product was dicalcium phosphate, which might have been the source of this element. In addition, in the other tablet products containing dicalcium phosphate, such as T1 (71 mg/d.d.), T6 (72 mg/d.d.), and T7 (79 mg/d.d.); considerable amounts of Ca were found (Table S2). The capsules and powders consisted mainly of beetroot ingredients. Their compositions were most similar to raw beetroot, as in the case of having K content as the main macroelement (266 mg/100 g of fresh conventional beetroot) [43]. The average Na content in a daily DS dose varied, with tablets having the lowest amount (2.3 mg/d.d.), capsules falling in the middle (5.3 mg/d.d.), and powders having the highest amount (61 mg/d.d.) (Table 2). A portion of fresh beetroot provided, on average, 35 mg/100 g of fresh weight (f.w.) of Na [43], which is less than some powdered DSs (P6: 41 mg/d.d., P9: 141 mg/d.d., P11: 190 mg/d.d., P13: 204 mg/d.d.) (Table S2). The mean content of Mg in the daily dose of the DSs also varied, with capsules having the lowest amount (1.3 mg/d.d.), tablets falling in the middle (1.9 mg/d.d.), and powders having the highest amount (17 mg/d.d.) (Table 2). A portion of fresh beetroot provided, on average, 22 mg/100 g f.w. of Mg [43].

#### Microminerals in DSs

Powders were characterized by a higher micromineral content per dose than what was found in tablets and capsules. Manganese was the most abundant micromineral (Table 2) in tablets (25 µg/d.d.), capsules (22 µg/d.d.), and powders (246 µg/d.d.). In the majority of cases, DSs provided less Mn than a portion of fresh beetroot (0.58 mg/100 g [43], 0.39 mg/100 g [52]). However, product P2 provided 1058 µg/d.d. and was the richest of all the samples analyzed (Table S2). Tablets supplied 0.78–10.8 µg/d.d. of Zn, capsules supplied 4.3–47 µg/d.d., and powders supplied 4.5–398 µg/d.d. (Table S2). A portion of fresh beetroot provided an average of 0.38 mg/100 g f.w. of Zn [43] and is comparable to some DSs in powder form (P2: 0.37 mg/d.d., P8: 0.40 mg/d.d.) (Table S2).

In contrast to our previous study, in which Ba was not detected above the LOQ (0.30 mg/kg) in DSs [43], capsules (49 µg/d.d.) and powders (517 µg/d.d.) were found to contain considerable amounts of Ba. A portion of fresh beetroot provided, on average, 0.175 mg/100 g f.w. of Ba [43], which was less than the average amount found in a portion of powder DSs (517 µg/d.d.).

Copper was determined to be above the LOQ in one tablet product (T1), four capsule products, and 11 powders (Table S2), while it was found in 75% of the conventional beetroot samples [43]. However, Cu was not detected above the LOQ in any organic beetroot samples [43].

### Realization of Dietary Recommendations

The nutritional and bioactive efficacy of the DSs was assessed based on the percentage of the RDA or AI for the chosen elements. The calculations were made for an average male aged 19–75 years, based on the nutritional recommendations for the Polish population [47]. For Na, K, and Mn, the AI values for these elements were applied. Dietary recommendations for men and women differ in the case of Mg (men: 420 mg/day, women: 320 mg/day), Fe (men: 10 mg/day, women: 18 mg/day), Zn (men: 11 mg/day, women: 8 mg/day), and Mn (men: 2.3 mg/day, women: 1.8 mg/day). However, this manuscript only refers to the recommendations for men to show a general tendency and make the results more transparent.

#### Contribution of the Analyzed Beetroot-Based DSs to Mineral Intake

Table S3 shows the specific results of the examined DSs’ fulfillment of dietary recommendations, whereas Table 2 shows a summary of the tablet, capsule, and powder DS groups. In all groups, the recommended DS portions led to a realization of the AI for K that did not exceed 4.1%. For capsules and tablets, the average realization of the AI for Na did not exceed 3.5%. Powders contained more Na, and the average realization of AI for this group was 4.1%. However, C7 (11% AI), C12 (9.4% AI), C13 (8.7% AI), P9 (9.4% AI), P11 (12.6% AI), and P13 (13.6% AI) provided considerable doses of Na in view of daily intake. For adults, the AI of Na is 1500 mg/day. It is worth noting that long-term excessive Na intake can lead to many serious health consequences, including hypertension, strokes, stomach cancer, and possibly esophageal cancer. It can also promote the development of osteoporosis and kidney stones, as well as obesity [47]. Capsules, tablets, and powders provided no more than 4.4% of the RDA for Mg and Ca.

The capsules and tablets delivered less than 1% of the AI for Mn. The powders constituted a considerable source of Mn, as the associated realization of AI amounted to 11%. The realization of the RDA for Zn was lower than 1.7%, while it ranged from 0.95 to 11% of the RDA for men for Mn. In Poland, the maximum DS level (ML) for Cr and Mo in DSs was set at 200 µg [53] and 350 µg [54], respectively, per daily portion. All of the studied products contained less than 10% of these values.

#### Fe-Enriched Products

**Fig. 1:**
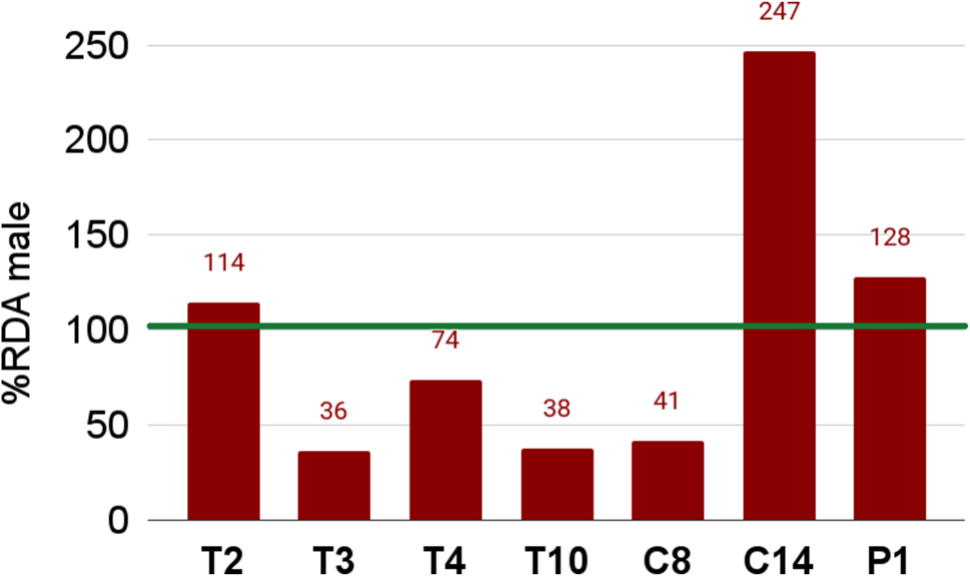
The realization of RDA for Fe for men according to recommendations for the Polish population (31)

Seven of the analyzed DSs were enriched with Fe fumarate or Fe gluconate (marked with * in Table S1). These organic compounds of Fe are the most popular forms of Fe in DSs [55], and they are highly bioavailable due to their easy ionization [56]. For Fe, the RDA for the Polish population is 10 mg/day for men [47]. The analyzed DSs contained 3.75–25 mg of elemental Fe (EDI), which provided an RDA realization (Figure 1) in the range of 36–247% for men. The encapsulated product C14 was the richest in Fe (247% of the RDA for men). The product contained 123% of the permissible Fe content in DSs, which is 20 mg in products that are not dedicated to pregnant women [57].

### Verification of Manufacturers’ Declarations Regarding Fe Content

Manufacturers declared that products T3, T4, and T10 contained 1.4 mg of Fe; that T2 and C8 contained 2.3 mg of Fe; that C14 contained 10 mg of Fe; and that P1 contained 14 mg of Fe (Table S1). Products T2, T4, and C8 did not meet the recommendations (Table 3) of the European Commission (EC) on setting tolerance limits for minerals contained on the labels, because the content of Fe was not in the range from −20 to +45% of the declared amount [48, 49].

**Table 3:** Compliance of the determined iron content with manufacturers’ declarations and guidelines

Sample	Declared Fe content (mg/d.u.)	Accepted minimum tolerance (−20%)	Accepted maximum tolerance (+45%)	Determined Fe content (mg/d.u.)	Compliance with the declaration (%)	Compliance with the guidelines
T2	2.3	1.8	3.3	5.7	248	No
T3	1.4	1.1	2.0	1.2	87	Yes
T4	1.4	1.1	2.0	2.5	176	No
T10	1.4	1.12	2.0	1.3	89	Yes
C8	2.8	2.24	4.1	2.1	74	No
C14	10	8.00	14.5	12.3	123	Yes
P1	14	11.07	20.1	12.8	92	Yes

*d.u.*, dosage unit

### Analysis of Results According to Regulatory Context

**Fig. 2:**
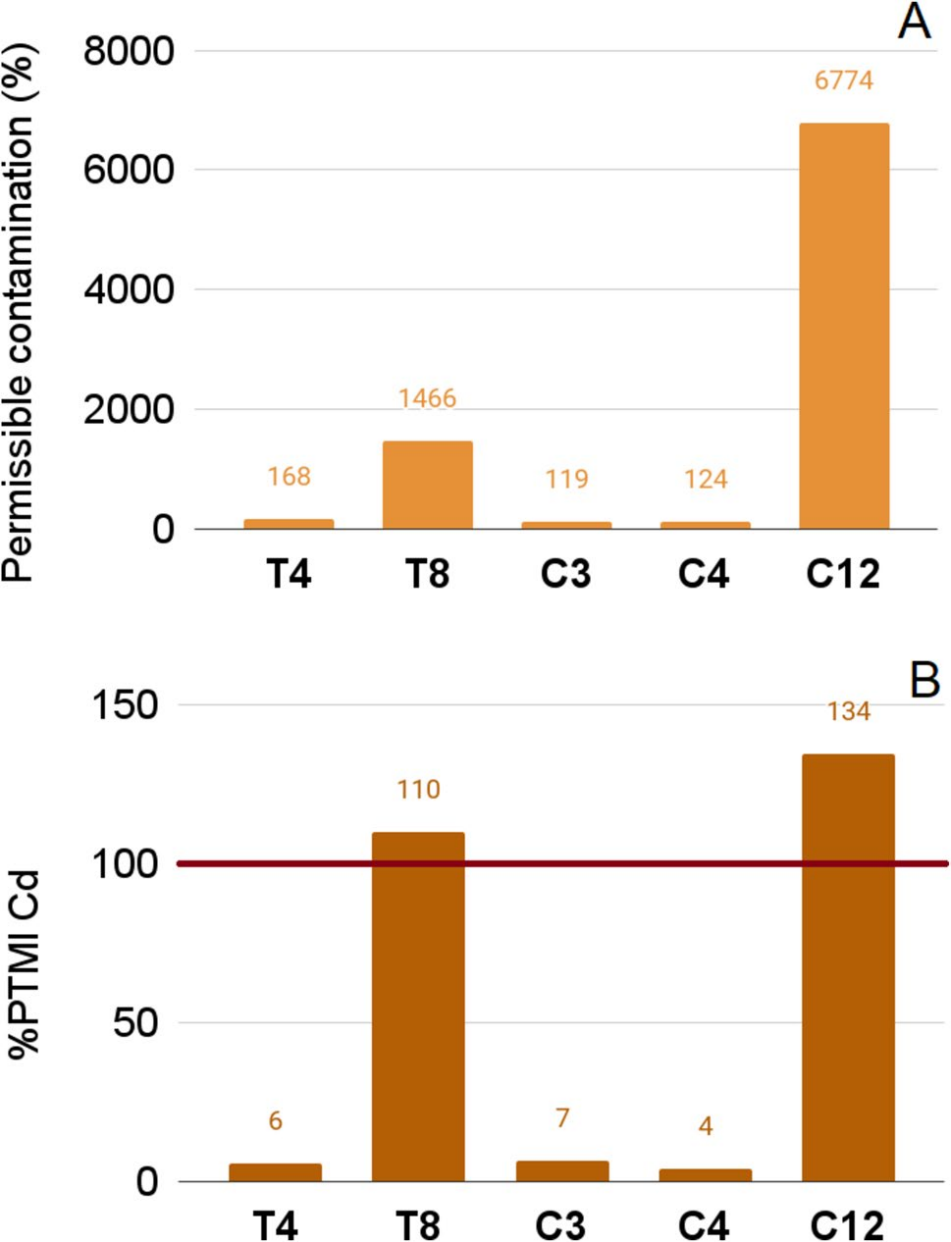
Determined content of Cd in DS samples expressed as a percentage of the maximum allowable level of its contamination (**A**). EDI value compared with PTMI value for Cd for an adult weighing 70 kg (1750 µg/70 kg/month) (**B**).

The permissible contamination limits of foodstuff are outlined by European Commission Regulations No. 1881/2006 and No. 629/2008 [28, 29]. The permissible contamination limit is 1 mg/kg for Cd [30] and 3 mg/kg for Pb [31]. Lead was not detected above the LOQ (<0.035 mg/kg) in any sample. Two products in tablet form (T4, T8) and 3 in capsule form (C3, C4, C12) were notably contaminated with Cd (Figure 2), and the amount of Cd in C12 was more than 6000 times the limit. This problem was also detected in our previous research, in which nearly 25% of the analyzed products were contaminated with Cd [43].

Research has shown that DSs containing Ca compounds [58], herbs or botanicals [59], or protein [60] as a major component do not pose a risk of Cd intoxication. However, there have been some reports of Cd contamination in fresh beetroots. Rusin et al. [61] showed that the Cd content in two beetroot samples exceeded the ML (203% and 670% of the ML) set by the EC for vegetables (0.1 mg/kg f.w. [62, 63]). Norton et al. [64] also found that the Cd content in one beetroot sample exceeded that of the ML (101% of ML).

The measurement of consumer risk might be expressed as the realization of PTMI for Cd. Two of the analyzed DSs exceeded 100% of the PTMI value (T8: 110% PTMI, C12: 134% PTMI). Hence, they should not be taken by consumers, even for a short period of time.

Barium poisoning happens rarely, but food is one of the possible sources of intoxication. The US Environmental Protection Agency (EPA) set an oral reference dose (RfD) of 14 mg of Ba/70 kg/day [65]. Products P6 (1.331 µg/d.d.), P2 (1.172 µg/d.d.), and P3 (1.085 µg/d.d.) provided a considerable dose of Ba and might pose a threat to consumers with long-term consumption [66]. Similarly, capsules (0.7–614 µg/d.d.) and powders (1.6–2420 µg/d.d.) contained considerable amounts of Al. Relating these portions to the PTWI for Al (140 mg/70 kg/ 7 days), 2 products might pose a risk for the consumer: P8 (11% PTWI) and P9 (12% PTWI).

Exposure to Cd compounds can result in an increased risk of carcinogenesis, especially in the prostate, kidneys, pancreas, and testicle [24, 67]. The intake of Ba compounds may result in gastrointestinal effects (vomiting, abdominal cramps, diarrhea), cardiovascular impairments (heart rhythm changes), or paralysis. The spectrum of effects on the human body depends on the rate of solubility in water and stomach acids, as well as the dose of Ba [66]. Beetroot, as a root vegetable, can accumulate toxic metals [20, 21]; thus, it is important to control finished products that include this ingredient. Moreover, Rusin et al. [61] pointed out that the highest concentrations of Cd and Pb are found in dried products.

### Statistical Analysis

#### Correlations

Spearman rank correlations were found at three levels of significance, i.e., *p* < 0.05, *p* < 0.01, and *p* < 0.001 with various correlation coefficients (*rs*). Positive correlations were obtained between the elements analyzed in the DSs containing beetroot and the natural raw material (beetroot). The correlations (*p* < 0.001) occurring between the elements in the database of DSs containing beetroot were as follows: K-Zn (*rs*=0.814), K-Mg (*rs*=0.566), K-Ba (*rs*=0.576), Fe-Mo (*rs*=0.557), Fe-Mg (*rs*=0.742), Fe-Mn (*rs*=0.709), Ca-Sr (*rs*=0.848), Ca-Ba (*rs*=0.555), Zn-Mg (*rs*=0.734), Zn-Mn (*rs*=0.636), Zn-Ba (*rs*=0.654), Mg-Mn (*rs*=0.634), Mg-Sr (*rs*=0.637), Mg-Ba (*rs*=0.722), Sr-Ba (*rs*=0.683).

The second Spearman rank correlation test concerned the natural raw beetroot materials and the DSs containing beetroot. The obtained elemental database showed the existence of the following correlations (*p* < 0.001): K-Zn (*rs*=0.745), K-Mg (*rs*=0.670), K-Mn (*rs*=0.496), K-Ba (*rs*=0.579), K-Al (*rs*=0.493), Fe-Zn (*rs*=0.512), Fe-Mg (*rs*=0.689), Fe-Mn (*rs*=0.705), Ca-Sr (*rs*=0.807), Ca-Ba (*rs*=0.527), Zn-Mg (*rs*=0.714), Zn-Mn (*rs*=0.686), Zn-Ba (*rs*=0.628), Mg-Mn (*rs*=0.675), Mg-Sr (*rs*=0.665), Mg-Ba (*rs*=0.710), Mg-Al (*rs*=0.605), Sr-Ba (*rs*=0.672), and Sr-Al (*rs*=0.479).

#### Kruskal–Wallis Test

The differences between pharmaceutical forms in view of elemental composition (category 1) were as follows (*p* < 0.05): K (H = 18.004; *p* = 0.000), Zn (H = 12.280; *p* = 0.002), Mg (H = 6.379; *p* = 0.041), Sr (H = 7.707; *p* = 0.021), and Ba (H = 10.081; *p* = 0.006). The Kruskal-Wallis test also revealed differences between the supplements enriched with Fe in view of certain elements (category 2) (*p* < 0.05), including Fe (H = 14.684; *p* = 0.000) and Mo (H = 14.059; *p* = 0.000). In category 3, significant differences were found between DSs classified according to the form of the main ingredient in view of the analyzed elements (*p* < 0.05): K (H = 13.171; *p* = 0.004), Fe (H = 12.660; *p* = 0.005), Zn (H = 12.980; *p* = 0.005), Mg (H = 13.201; *p* = 0.004), Mn (H = 10.111; *p* = 0.017), and Ba (H = 9.304; *p* = 0.025). Using the analyzed database of raw material (beetroot) [35] and DSs containing beetroot, the Kruskal-Wallis test showed the following differences (*p* < 0.05): K (H = 21.881; *p* = 0.002), Fe (H = 14.860; *p* = 0.005), Mg (H = 21.058; *p* = 0.000), Mn (H = 14.820; *p* = 0.005), and Ba (H = 15.087; *p* = 0.004).

#### Post-hoc Dunn’s Test

A post-hoc Dunn’s test was used to identify which groups were significantly related. The Dunn test was conducted for the same data categories as the Kruskal-Wallis test and at three levels of significance (i.e., *p* < 0.05, *p* < 0.01, and *p* < 0.001).

The results of the Dunn test on the data regarding DSs containing beetroot were classified according to their pharmaceutical forms (tablet, capsule, powder), as shown in Table 4. The most significant relationships were obtained between tablet and powder forms for K and Zn. For some samples of Fe-enriched DSs, a significant relationship was found between Fe and Mo (*p* < 0.001) when compared to non-enriched products.

**Table 4:** Results of the Dunn’s test for the data matrix concerning the pharmaceutical form of dietary supplements (significance values in superscript)

	Tablet	Capsule	Powder
Tablet	-		Ba^0.005^, K^0.000^, Zn^0.001^
Capsule		-	K^0.047^, Zn^0.042^, Mg^0.041^, Sr^0.017^
Powder	Ba^0.005^, K^0.000^, Zn^0.002^	K^0.047^, Zn^0.042^, Mg^0.041^, Sr^0.017^	-

A post-hoc test was also carried out for DS samples in terms of the form of the main ingredients they contained. Dunn’s test revealed significant relationships, which are presented in Table 5. The test revealed significant interrelationships for Zn and Mg between the extract and the beetroot powder.

**Table 5:** Results of the Dunn’s test for the data matrix concerning the main ingredient of dietary supplements (significance values in superscript).

	Extract	Concentrate	Beetroot powder	Dried juice
Extract	-	Fe^0.016^, Mo^0.041^	Ba^0.044^, K^0.011^, Fe^0.040^, Mg^0.003^, Zn^0.012^	
Concentrate	Fe^0.016^, Mo^0.041^	-		
Beetroot powder	Ba^0.044^, K^0.011^, Fe^0.040^, Mg^0.003^, Zn^0.012^		-	
Dried juice				-

The raw beetroot data published by Brzezińska-Rojek et al. [43], as well as the data from the supplements analyzed in this study, were used in the Dunn test. The results of the post hoc test are presented in Table 6. The strongest relationships were obtained for Mg and K between the raw beetroot and beetroot extracts in the DSs.

**Table 6:** Results of the Dunn’s test for the data matrix concerning the main ingredient of dietary supplements and raw beetroot (significance values in superscript).

	Extract	Concentrate	Beetroot powder	Dried juice	Beetroot raw material
Extract	-	Fe^0.013^	K^0.047^, Zn^0.041^, Ba^0.016^, Mg^0.01^		Mn^0.020^, Al^0.006^, K^0.000^, Mg^0.000^
Concentrate	Fe^0.013^	-			K^0.034^
Beetroot powder	K^0.047^, Zn^0.041^, Ba^0.016^, Mg^0.01^		-		
Dried juice				-	
Beetroot raw material	Mn^0.020^, Al^0.006^, K^0.000^, Mg^0.000^	K^0.034^			-

#### Factor Analysis

**Fig. 3:**
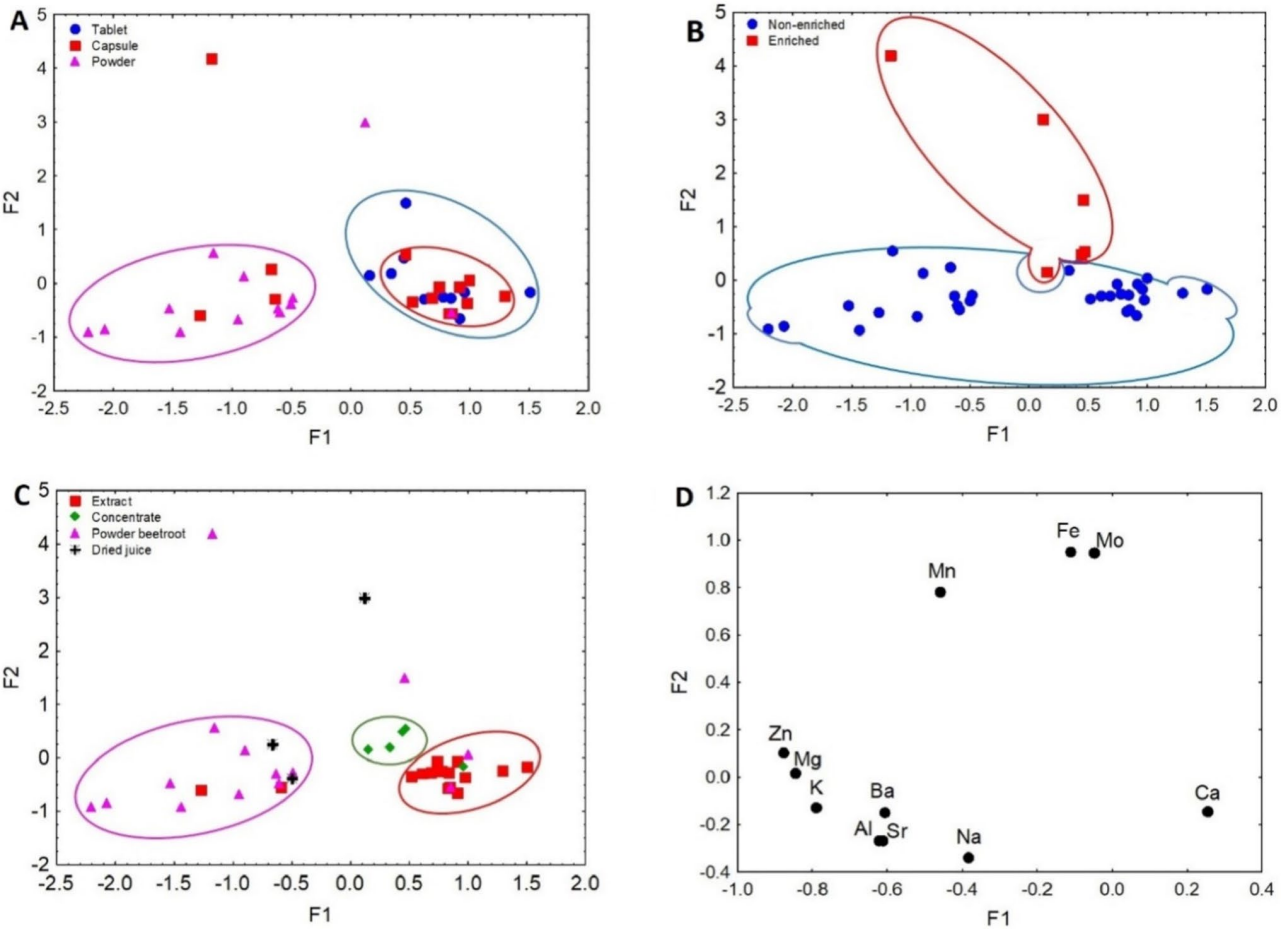
**A** Scatterplot of object samples of two factors of all dietary supplements in view of their pharmaceutical form. **B** Scatterplot of object samples of two factors of all dietary supplements in view of their enrichment. **C** Scatterplot of object samples of two factors of all dietary supplements in view of a form of the main ingredient. **D** Scatterplot of loadings for elements in all the analyzed samples.

**Fig. 4:**
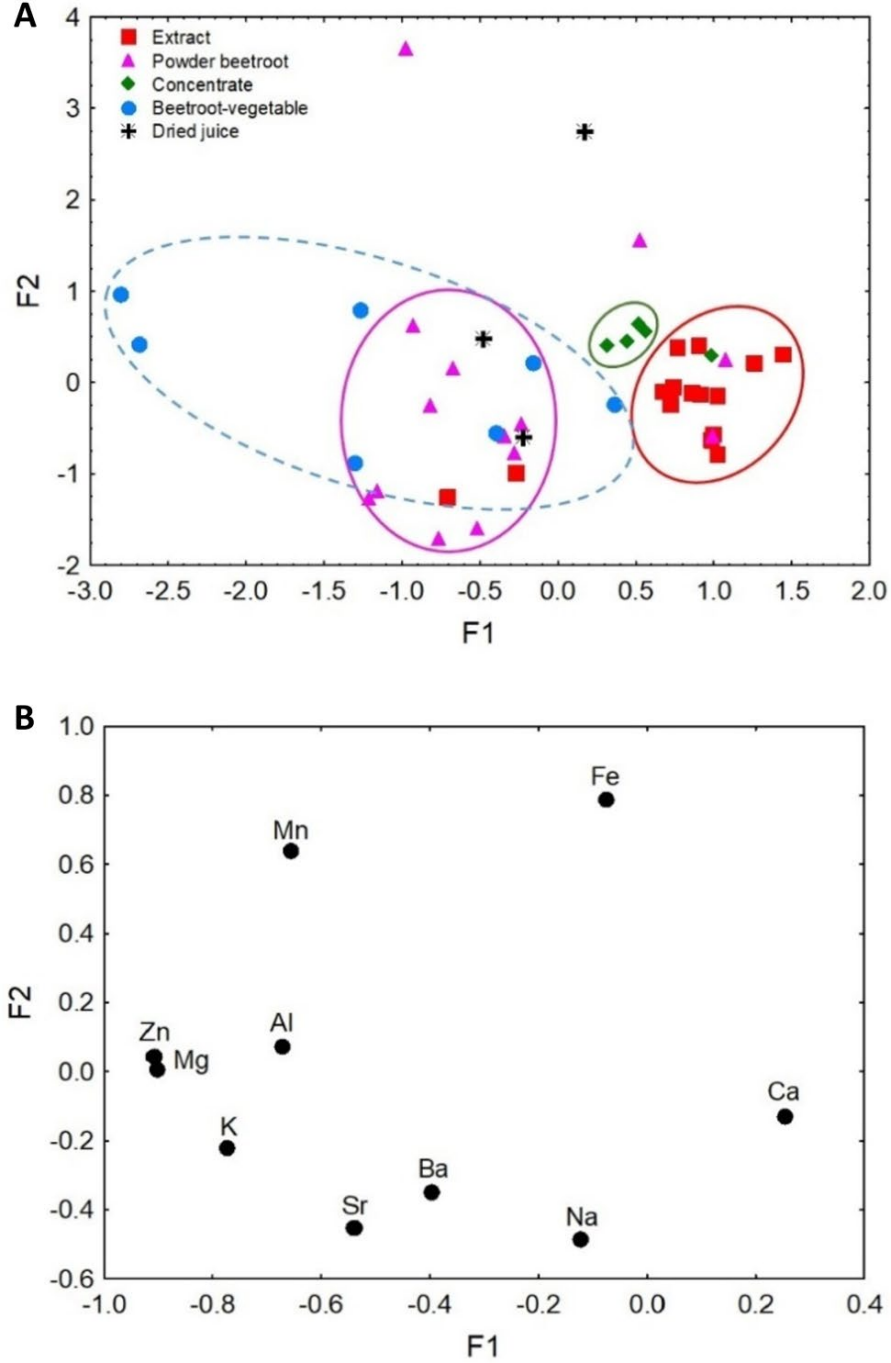
**a** Scatterplot of object samples of two factors of all dietary supplements and beetroot samples. **b** Scatterplot of loadings for elements in all the analyzed samples.

Bartlett’s test of sphericity was performed for a database of DS containing beetroot samples. The results of this test were found to be significant (*p*=0.000). The Kaiser-Meyer-Olkin Measure of Sampling Adequacy (KMO) was 0.589, which according to the criteria [68] allows us to perform the analysis. The first-factor analysis was performed on all the data of the analyzed DSs containing beetroot, classified according to pharmaceutical form, Fe enrichment, and the form of the main ingredient in the DS (Figure 3A–D). The second analysis, which is presented in Figure 4a and b, aimed to show the distribution of the DS samples containing beetroot compared to that of raw beetroot (data taken from Brzezińska-Rojek et al. [43]).

For the data divided by pharmaceutical form (tablets, capsules, and powders), the value of the first factor (F1) explained 33.54% of the variance, while the second factor (F2) explained 24.92%. Both factors cumulatively explained 58.46% of the total variance, where the eigenvalues for F1 and F2 were 3.69 and 2.74, respectively. As can be observed in Figure 3A, F1 is responsible for the differentiation of DS samples in powder form from those in tablet form. In the case of capsules, some were attributed to the powder group, which may suggest that they contained the same powders used in the powder-form supplements. The low values of the F1 factor correspond to the powders, which were described by the elements Na, Sr, Ba, Al, K, Mg, Zn, and Mn. High F1 values corresponding to DSs in tablet form were described by Ca, Mo, and Fe (Figure 3D). The performed analysis not only makes it possible to differentiate DS samples based on their pharmaceutical form but also to show similarities between the content of capsules and that of powders.

The distribution of the studied products by pharmaceutical form, which is shown in Figure 3A, is similar to that of DSs enriched in Fe (Figure 3B). The F2 factor was mainly responsible for the separation of the samples according to product enrichment. High F2 values correspond to enriched samples, as described by Mn, Fe, and Mo (Figure 3D). Because DSs are made of plant material (i.e., beetroot), it is probable that the assemblages of Mn, Fe, and Mo next to each other indicate their interaction. In Vigani et al.’s [69] study on cucumbers, it was confirmed that the presence of Mo influences increased Fe uptake in plants because most molybdenum-containing enzymes in plants require Fe-containing redox groups [70]. Lawson-Wood et al. [71] showed that Mn and Fe being at the highest levels results in both elements being taken up more efficiently by plants. Low F2 values were responsible for the diversification of non-enriched samples, characterized by Zn, Mg, K, Ba, Al, Sr, Na, and Ca. Factor analysis of the DSs under study allowed for the identification of samples enriched in Fe.

In the next analysis, F1 diversified the DS samples containing beetroot extract, concentrate, and beetroot powder (Figure 3C). The high F1 values, described by Fe, Mo, and Ca, corresponded with the DS samples containing beetroot in the concentrate and extract forms. The presence of Fe as a descriptor is related to the enrichment of this group, while Ca is probably derived from the water from which the concentrate was prepared. On the other hand, samples containing beetroot powder in their composition had low F1 values and were characterized by Mn, Zn, Mg, K, Ba, Al, Sr, and Na. The presence of a form of dried beetroot juice in this group, as well as several extract samples, may indicate mislabeling of the product. The chemometric analysis distinguished samples of DSs containing beetroot in terms of the form of the main ingredient used but also verified the information given on the product label.

Bartlett’s test of sphericity that was conducted for DS data containing beetroot and raw beetroot material [35] showed a significance of *p*=0.000, and a KMO score amounted to 0.562, so the data was subjected to FA analysis [68]. This factor analysis aimed to assess the similarity between the natural raw material (raw beetroot) and DS samples containing beetroot. The raw material data were taken from content published by Brzezińska-Rojek et al. [43]. The F1 value explained 36.67% of the variance, while the F2 value explained 16.63%. Together, the two factors explained 53.30% of the total variance. The eigenvalues of F1 and F2 were 3.67 and 1.66, respectively. High F1 values were responsible for the distribution of samples containing extracts and beetroot concentrate in the DS. The low F1 values showed a close relationship between the beetroot powder and the raw beetroot samples, suggesting that the beetroot powder may have been formed from the natural raw material. It can be assumed that, as the manufacturer specifies on the product label, the beetroot powder is indeed the natural raw material in powder form. The samples of beetroot powder and raw material were described by elements such as Na, Ba, Sr, K, Al, Mg, Zn, and Mn (i.e., elements of soil origin). The use of natural raw beetroot data in FA, combined with data from DSs containing beetroot, made it possible to demonstrate the authenticity and quality of the products analyzed.

The factor analyses presented in Figure 3A, B, C, and D show the joint influence of the pharmaceutical form, the Fe enrichment of the product, and the form of the main ingredient present in the DS on the identification and verification of DSs. Additionally, the factor analysis of the DSs and samples of the natural raw material (beetroot) provide important information about the authenticity of the ingredient used in the supplement (Fig. 4a, b) and the quality of the final product.

## Conclusion

In this research, 37 beetroot-based DSs were assessed for safety and health value based on mineral composition and Al, Ba, Cd, and Pb content. In general, DSs in powder form contained higher doses of elements than DSs in capsule and tablet forms. The exception was Fe-enriched products, mainly available in capsule or tablet form, which provided a significant dose of Fe because of RDA fulfillment. The capsule product C14 contained the highest dose of Fe (247% of the RDA for men). Some DSs supplied considerable doses of Na; thus, they should be considered when estimating the daily consumption of Na. The study revealed that 5 products were considerably contaminated with Cd, which translated into notable PTMI realizations. In the majority of cases, DSs provide low health value because of their mineral delivery to consumers. Moreover, DSs contaminated with Cd might pose a risk to consumers. Factor analysis was useful in differentiating beetroot-based products based on their pharmaceutical form and the type of beetroot preserves they used. Moreover, it allowed for an evaluation of the authenticity and safety of the examined products.

In conclusion, in the majority of cases, DSs provided fewer minerals than a 100 g portion of fresh beetroot. Some of the available beetroot-based products might pose a risk for consumers due to their accumulative abilities and contamination with toxic elements. The research revealed that there is a strong need for the evaluation of DSs which are launched and for a requirement that quality certificates be provided for finished products.

### Supplementary Information

Below is the link to the electronic supplementary material.

Supplementary file1 (DOCX 56 KB) The following are available online at [link], Table S1 Full characteristics of the analyzed beetroot-based dietary supplements according to information on the package (* iron-enriched products). Table S2 The content of elements in beetroot-based DSs (xm ± U, (k = 2)). Table S3 Results of the realization of dietary recommendation (%) for selected elements according to Jarosz et al. [47] by a daily portion of DSs (EDI). Table S4 Comparison of EDI of DSs with chosen toxicological parameters. Results are expressed as percentage of realization.

## Data Availability

All data generated or analyzed during this study are included in this published article and its supplementary information files.

## References

[1] Milton-Laskibar I, Alfredo Martínez J, Portillo MP (2021). Current knowledge on beetroot bioactive compounds: role of nitrate and betalains in health and disease. Foods.

[2] El-Sohaimy SA, Abdo E, Shaltout O (2020). Nutritional evaluation of beetroots (Beta vulgaris L.) and its potential application in a functional beverage. Plants.

[3] Gawęda M (1998). The role of some components of the substrate in reducing the accumulation of lead by selected species of root vegetables and leafy and their importance for preserving biological value of plants. Zesz Nauk Akad Rol w Krakowie.

[4] Odoh UE, Ezugwu CO, Okoro EC (2012). Quantitative phytochemical, proximate/nutritive composition analysis of Beta vulgaris Linnaeus (Chenopodiaceae). Planta Med.

[5] Sharma KD, Karki S, Thakur NS, Attri S (2012). Chemical composition, functional properties and processing of carrot-a review. J Food Sci Technol.

[6] Chhikara N, Kushwaha K, Sharma P (2019). Bioactive compounds of beetroot and utilization in food processing industry: a critical review. Food Chem.

[7] Belitz H-D, Grosch W, Schieberle P, Belitz H-D, Grosch W, Schieberle P (2004). Minerals. Food Chemistry.

[8] Shrimanker I, Bhattarai S (2022) Electrolytes. In: StatPearls. StatPearls Publishing, Treasure Island (FL). https://pubmed.ncbi.nlm.nih.gov/31082167/ Accessed 24 Feb 2023 31082167

[9] Pohl HR, Wheeler JS, Murray HE (2013) Sodium and potassium in health and disease. In: Sigel A, Sigel H, Sigel R (eds) Interrelations between Essential Metal Ions and Human Diseases. Metal Ions in Life. Springer, Dordrecht, pp 29–47 10.1007/978-94-007-7500-8_2 24470088

[10] Brini M, Ottolini D, Calì T, Carafoli E (2013) Calcium in health and disease. In: Sigel A, Sigel H, Sigel R (eds) Interrelations between Essential Metal Ions and Human Diseases. Metal Ions in Life. Springer, Dordrecht, pp 81–137 10.1007/978-94-007-7500-8_4 24470090

[11] Romani AMP (2013) Magnesium in health and disease. In: Sigel A, Sigel H, Sigel R (eds) Interrelations between Essential Metal Ions and Human Diseases. Metal Ions in Life Sciences. Springer, Dordrecht, pp 49–79 10.1007/978-94-007-7500-8_3 24470089

[12] Barbagallo M, Veronese N, Dominguez LJ (2021). Magnesium in aging, health and diseases. Nutrients.

[13] Avila DS, Puntel RL, Aschner M (2013) Manganese in health and disease. In: Sigel A, Sigel H, Sigel R (eds) Interrelations between Essential Metal Ions and Human Diseases. Metal Ions in Life Sciences. Springer, Dordrecht, pp 199–227 10.1007/978-94-007-7500-8_7 PMC6589086 24470093

[14] Scheiber I, Dringen R, Mercer JFB (2013) Copper: effects of deficiency and overload. In: Sigel A, Sigel H, Sigel R (eds) Interrelations between Essential Metal Ions and Human Diseases. Metal Ions in Life Sciences. Springer, Dordrecht, pp 359–387 10.1007/978-94-007-7500-8_11 24470097

[15] Maret W (2013) Zinc and human disease. In: Sigel A, Sigel H, Sigel R (eds) Interrelations between essential metal ions and human diseases. Metal Ions in Life Sciences. Springer, Dordrecht, pp 389–414 10.1007/978-94-007-7500-8_12 24470098

[16] Hider RC, Kong X (2013) Iron: effect of overload and deficiency. In: Sigel A, Sigel H, Sigel R (eds) Interrelations between Essential Metal Ions and Human Diseases. Metal Ions in Life Sciences. Springer, Dordrecht, pp 229–294 10.1007/978-94-007-7500-8_8 24470094

[17] Chen P, Bornhorst J, Aschner M (2018). Manganese metabolism in humans. Front Biosci - Landmark.

[18] Chasapis CT, Ntoupa PSA, Spiliopoulou CA, Stefanidou ME (2020). Recent aspects of the effects of zinc on human health. Arch Toxicol.

[19] Abbaspour N, Hurrell R, Kelishadi R (2014). Review on iron and its importance for human health. J Res Med Sci.

[20] Saletnik B, Zaguła G, Bajcar M, Puchalski C (2016). Accumulation of cadmium, lead and mercury in seedlings of selected sugar beet varieties as a result of simulated soil contamination. J Microbiol Biotechnol Food Sci.

[21] Ćwieląg-Drabek M, Piekut A, Gut K, Grabowski M (2020). Risk of cadmium, lead and zinc exposure from consumption of vegetables produced in areas with mining and smelting past. Sci Rep.

[22] Poniedziałek M, Sękara A, Ciura J, Jędrzejczyk E (2005). Nickel and manganese accumulation and distribution in organs of nine crops. Folia Hortic.

[23] Dziubanek G, Baranowska R, Ćwieląg-Drabek M (2017). Cadmium in edible plants from Silesia, Poland, and its implications for health risk in populations. Ecotoxicol Environ Saf.

[24] IARC Working Group on the Evaluation of Carcinogenic Risks to Humans. (2012) Cadmium and cadmium compounds. In: IARC Monographs on the Evaluation of Carcinogenic Risks to Humans. International Agency for Research on Cancer, Lyon, FR, pp 121–145 10.1002/14356007.a04_499

[25] Organization WH (2010) Exposure to cadmium: a major public health concern. In: Prev. Dis. through Heal. Environ. https://www.who.int/publications/i/item/WHO-CED-PHE-EPE-19-4-3. Accessed 2 Oct 2021

[26] Gajda-Wyrębek J, Jarecka J, Dmitruk M (2021). Monitoring survey of nitrate content in beetroot, radish and cabbage in Poland. Rocz Panstw Zakl Hig.

[27] WHO (2021) Exposure to lead: a major public health concern. In: Prev. Dis. through Heal. Environ. https://www.who.int/publications/i/item/9789240037656. Accessed 21 Jul 2023

[28] The Commission of the European Communities (2006) Commission Regulation (EC) No 1181/2006 of 19 December 2006 setting maximum levels for certain contaminants in foodstuffs. Off J Eur Union 5–24. https://eur-lex.europa.eu/legal-content/EN/TXT/PDF/?uri=CELEX:02006R1881-20230326. Accessed 21 Jul 2023

[29] The Commission of the European Communities (2008) Commission regulation (EC) No 629/2008 of 2 July 2008 amending Regulation (EC) No 1881/2006 setting maximum levels for certain contaminants in foodstuffs. Off J Eur Union 6–9. https://eur-lex.europa.eu/LexUriServ/LexUriServ.do?uri=OJ:L:2008:173:0006:0009:EN:PDF. Accessed 21 Jul 2023

[30] JECFA Evaluations of the Joint FAO/WHO Expert Committee on Food Additives (JECFA) on cadmium. https://apps.who.int/food-additives-contaminants-jecfa-database/Home/Chemical/1376. Accessed 24 Feb 2023

[31] JECFA Evaluations of the Joint FAO/WHO Expert Committee on Food Additives (JECFA) on lead. https://apps.who.int/food-additives-contaminants-jecfa-database/Home/Chemical/3511. Accessed 24 Feb 2023

[32] Brzezińska J, Grembecka M (2021). Dietary supplements – specific food. Adv Hyg Exp Med.

[33] Champagne AB, Emmel KV (2011). Rapid screening test for adulteration in raw materials of dietary supplements. Vib Spectrosc.

[34] Biesterbos JWH, Sijm DTHM, van Dam R, Mol HGJ (2019). A health risk for consumers: the presence of adulterated food supplements in the Netherlands. Food Addit Contam - Part A Chem Anal Control Expo Risk Assess.

[35] da Justa Neves DB, Caldas ED (2015). Dietary supplements: international legal framework and adulteration profiles, and characteristics of products on the Brazilian clandestine market. Regul Toxicol Pharmacol.

[36] Czepielewska E, Makarewicz-Wujec M, Różewski F (2018). Drug adulteration of food supplements: a threat to public health in the European Union?. Regul Toxicol Pharmacol.

[37] The Seym of the Republic of Poland (2006) Dz. U. 2006 Nr 171 poz. 1225. Act of August 25, 2006 on food and nutrition safety (as amended). Internet System of Legal Acts, Poland. https://isap.sejm.gov.pl/isap.nsf/DocDetails.xsp?id=wdu20061711225. Accessed 21 Jul 2023

[38] The Seym of the Republic of Poland (2023) Draft Law on Amendments to the Law on Food and Nutrition Safety in Poland. https://legislacja.rcl.gov.pl/projekt/12367901/katalog/12941901#12941901. Accessed 21 Jul 2023

[39] Nizioł-Lukaszewska Z, Gawęda M (2016). Influence of cultivar on the content of selected minerals in red beet roots (Beta vulgaris L.). Folia Hortic.

[40] Székely D, Furulyás D, Stéger-Máté M (2019) Investigation of mineral and vitamin C contents in different parts of beetroots (Beta vulgaris L.). Not Bot Horti Agrobot Cluj-Napoca 47: 10.15835/nbha47311394

[41] Kale R, Sawate A, Kshirsagar R (2018). Studies on evaluation of physical and chemical composition of beetroot (Beta vulgaris L.). Int J Chem Stud.

[42] Jurowski K, Krośniak M, Fołta M (2019). The analysis of Cu, Mn and Zn content in prescription food for special medical purposes and modified milk products for newborns and infants available in Polish pharmacies from toxicological and nutritional point of view. J Trace Elem Med Biol.

[43] Brzezińska-Rojek J, Rutkowska M, Brzezicha J (2022). Mineral composition of dietary supplements-analytical and chemometric approach. Nutrients.

[44] Huber L (2003) Validation of analytical methods and processes. In: Nash RA, Wachter AH (eds) Pharmaceutical Process Validation, CRC Press: Boca Raton, FL, USA

[45] Jurowski K, Krośniak M, Fołta M (2019). The toxicological analysis of Ni and Cr in prescription food for special medical purposes and modified milk products for babies in infancy available in pharmacies in Poland. Biol Trace Elem Res.

[46] Jurowski K, Krośniak M, Fołta M (2019). The toxicological analysis of lead and cadmium in prescription food for special medical purposes and modified milk products for newborns and infants available in Polish pharmacies. J Trace Elem Med Biol.

[47] Jarosz M, Rychlik E, Stoś K, Charzewska J (2020) Normy żywienia dla populacji Polski i ich zastosowanie. Narodowy Instytut Zdrowia Publicznego – Państwowy Zakład Higieny, Warszawa

[48] Europe Food Supplements (2014) Setting of tolerances for nutrient vaues declared on a label. Guidance for food supplements. https://foodsupplementseurope.org/wp-content/themes/fse-theme/documents/publications-and-guidelines/fse-setting-of-tolerances-for-nutrient-values-declared-on-a-label.pdf. Accessed 24 Feb 2023

[49] Wawrzyniak A, Przybyłowicz K, Wądołowska L (2021). Statement of the Committee of Human Nutrition Science of the Polish Academy of Sciences on the use of dietary supplements containing vitamins and minerals by adults. Rocz Panstw Zakl Hig.

[50] United States Pharmacopeial, Convention (2021) <2232> Elemental contaminants in dietary supplements. In: States Pharmacopeia and National Formulary (USP 43-NF 38). United States Pharmacopeial Convention, Rockville, MD, USA

[51] Szefer P (2006) Chemometric techniques in analytical evaluation of food quality. In: Szefer P, Nriagu J (eds) Mineral Components in Foods. CRC Press – Taylor & Francis, Boca Raton, FL, USA, pp 69–121

[52] Lisiewska Z, Kmiecik W, Gębczyński P (2006). Effects on mineral content of different methods of preparing frozen root vegetables. Food Sci Technol Int.

[53] Dietary Supplements Panel (2020) Resolution No. 4/2020 of the Dietary Supplements Panel of 25 October 2019 on expressing an opinion on the maximum amount of chromium in the recommended daily dose in dietary supplements. Chief Sanitary Inspector. https://www.gov.pl/attachment/0ed6c316-4dfb-4f30-b50a-f0f0a82cfb69. Accessed 21 Jul 2023

[54] Dietary Supplements Panel (2021) Resolution No. 8/2021 of the Dietary Supplements Panel of 28 October 2021 on expressing an opinion on the maximum dose of molybdenum in the recommended daily dose in dietary supplements. Chief Sanitary Inspector. https://www.gov.pl/attachment/0cde167b-eaa9-433c-9854-1209032d588b. Accessed 21 Jul 2023

[55] Fairweather-Tait SJ, Teucher B (2002). Iron and calcium bioavailability of fortified foods and dietary supplements. Nutr Rev.

[56] Lönnerdal B, Hernell O (2013) Iron: physiology, dietary sources, and requirements. In: Encyclopedia of Human Nutrition. Academic Press, vol 3, pp 39–46. 10.1016/B978-0-12-375083-9.00164-1

[57] Dietary Supplements Panel (2019) Resolution No. 20/2019 of the dietary supplements panel of 13 December 2019 on expressing an opinion on the maximum dose of iron in the recommended daily dose in dietary supplements. Chief Sanitary Inspector. https://www.gov.pl/attachment/ea175951-4f90-400e-b063-a66bd8689344. Accessed 21 Jul 2023

[58] Kim M (2004). Mercury, cadmium and arsenic contents of calcium dietary supplements. Food Addit Contam.

[59] Dolan SP, Nortrup DA, Bolger PM, Capar SG (2003). Analysis of dietary supplements for arsenic, cadmium, mercury, and lead using inductively coupled plasma mass spectrometry. J Agric Food Chem.

[60] Bandara SB, Towle KM, Monnot AD (2020). A human health risk assessment of heavy metal ingestion among consumers of protein powder supplements. Toxicol Reports.

[61] Rusin M, Domagalska J, Rogala D (2021). Concentration of cadmium and lead in vegetables and fruits. Sci Rep.

[62] European Commission (2015). Commission Regulation (EU) 2015/ 1005 25 June 2015 amending Regulation (EC) No 1881/2000 as regards maximum levels of lead in certain foodstuffs. Off J Eur Union.

[63] European Commission (2014). Commission Regulation (EU) No 488/2014 of 12 May 2014 amending Regulation (EC) No 1881/2006 as regards maximum levels of cadmium in foodstuffs. Off J Eur Union.

[64] Norton GJ, Deacon CM, Mestrot A (2015). Cadmium and lead in vegetable and fruit produce selected from specific regional areas of the UK. Sci Total Environ.

[65] U.S. Environmental Protection Agency, Integrated Risk Information System (IRIS) (2005) Barium and compounds. http://www.epa.gov/iris/backgrd.html. Accessed 28 Mar 2023

[66] Minns A (2018) Barium. In: Olson K, Anderson I, Benowitz N, et al (eds) Poisoning & drug overdose, 7e ed. McGraw Hill Medical, New York, NY, USA, pp 109–110

[67] Rehman K, Fatima F, Waheed I, Akash MSH (2018). Prevalence of exposure of heavy metals and their impact on health consequences. J Cell Biochem.

[68] Field A (2017). Discovering statistics using IBM SPSS statistics.

[69] Vigani G, Di Silvestre D, Agresta AM (2017). Molybdenum and iron mutually impact their homeostasis in cucumber (Cucumis sativus) plants. New Phytol.

[70] Bittner F (2014). Molybdenum metabolism in plants and crosstalk to iron. Front Plant Sci.

[71] Lawson-Wood K, Jaafar M, Felipe-Sotelo M, Ward NI (2021). Investigation of the uptake of molybdenum by plants from Argentinean groundwater. Environ Sci Pollut Res.

