# Supplementary material for: Evaluation of the Safety and Potential Benefits of Beetroot-Based Dietary Supplements According to Their Elemental Composition
Source: Biol Trace Elem Res. 2023 Oct 7;202(7):3318–32. doi: 10.1007/s12011-023-03902-x (PMC11074222; doi:10.1007/s12011-023-03902-x)
Supplement: Supplementary file 1 — Supplementary file1 (DOCX 56 KB) The following are available online at [link], Table S1 Full characteristics of the analyzed beetroot-based dietary supplements according to information on the package (* iron-enriched products). Table S2 The content of elements in beetroot-based DSs (xm ± U, (k = 2)). Table S3 Results of the realization of dietary recommendation (%) for selected elements according to Jarosz et al. [47] by a daily portion of DSs (EDI). Table S4 Comparison of EDI of DSs with chosen toxicological parameters. Results are expressed as percentage of realization. [file 12011_2023_3902_MOESM1_ESM.docx]

Table S1 Full characteristics of the analyzed beetroot-based dietary supplements according to information on the package (* iron-enriched products).

| Form | Code | Number of dosage units or doses | The content of beetroot preserves/dosage unit | Declared weight of the dosage unit or dose1 (g) | Recommen- dation | Origin country |
| --- | --- | --- | --- | --- | --- | --- |
| tablets | T1 | 100 | 132.375 mg of dicalcium phosphate; 132.375 mg of microcrystalline cellulose; 80 mg of beetroot extract; 2.25 mg vegetable magnesium stearate | 0.35 | 2 tab. | UK |
|  | T2* | 60 | 500 mg of powdered beetroot; maltodextrin; 40 mg of vitamin C; 7 mg of iron (II) fumarate; bulking agent: sorbitols; anti-caking agents: magnesium salts of fatty acids, silicon dioxide | 0.63 | 2x1 tab. | PL |
|  | T3* | 60 | 500 mg of beetroot concentrate; 20 mg of vitamin C; 12 mg of iron (II) gluconate; starch; anti-caking agent: magnesium salts of fatty acids, silicon dioxide | 0.65 | 3x1 tab. | PL |
|  | T4* | 60 | 500 mg of beetroot concentrate; 20 mg of vitamin C; 12 mg of iron (II) gluconate; starch; anti-caking agent: magnesium salts of fatty acids, silicon dioxide | 0.65 | 3x1 tab. | PL |
|  | T5 | 60 | 500 mg of beetroot concentrate; 1 mg of vitamin B6; 1.25 mg of vitamin B12; anti-caking agents: magnesium salts of fatty acids, silicon dioxide | 0.50 | 3x2 tab. | PL |
|  | T6 | 120 | 350 mg of beetroot extract 20; binder: dicalcium phosphate; emulsifier: microcrystalline cellulose; stabilizer: magnesium salts of fatty acids | 0.35 | 1x2 tab. with a meal | UK |
|  | T7 | 120 | 350 mg of beetroot extract 20:1; binder: dicalcium phosphate; emulsifier: microcrystalline cellulose; stabilizer: magnesium salts of fatty acids | 0.35 | 1x1 tab. | UK |
|  | T8 | 100 | 1000 mg of beetroot extract; bulking agent: microcrystalline cellulose; anti-caking agent: stearic acid, magnesium stearate; stabilizer and solubiliser: sodium croscarmellose | 1.46 | 3x1 tab. | USA |
|  | T9 | 90 | 300 mg of beetroot extract; bulking agents: dicalcium phosphate, microcrystalline cellulose; anti-caking agents: stearic acid, silicon dioxide, magnesium stearate; glazing agents: hydroxypropyl methylcellulose, glycerin, carnauba wax | 0.821 | 3x1 tab. | UK |
|  | T10* | 60 | 488 mg of beetroot concentrate; 20 mg of vitamin C; 12 mg of iron gluconate; starch; anti-caking agent: magnesium salts of fatty acids; silicon dioxide | 0.65 | 3,0 | PL |
| capsules | C1 | 100 | 500 mg of powdered beetroot; magnesium stearate; gelatine capsule | 0.507 | 3X2 caps | USA |
|  | C2 | 60 | 700 mg of organic beetroot extract with maltodextrin; vegetable capsule shell | 0.714 | 1x2 caps.  with meal | UK |
|  | C3 | 100 | 500 mg of powdered beetroot; vegetable capsules; cellulose; silica; magnesium stearate | 0.539 | 3x2 caps.  with meal | USA |
|  | C4 | 90 | 500 mg of beetroot extract; cellulosan; silicon dioxide; vegetable fatty; vegetable mineral salts | 0.625 | 3x1 caps.  with meal | USA |
|  | C5 | 60 | 450 mg of beetroot extract; bulking agent: microcrystalline cellulose; shells: hydroxypropyl methylcellulose | 0.584 | 2x1 caps. | USA |
|  | C6 | 90 | 500 mg of beetroot extract (5:1); bulking agents: maltodextrin, microcrystalline cellulose; vegetable capsule shell: hydroxypropyl methylcellulose; anti-caking agents: silicon dioxide, vegetable magnesium stearate | 0.628 | 1x1 caps. | USA |
|  | C7 | 60 | 550 mg of Beta vulgaris extract 4:1; pullulan capsule | 0.614 | 1x2 caps. | PL |
|  | C8* | 90 | 438.5 mg of dried juice concentrate; 38 mg of vitamin C; 2.8 mg of iron; capsule shell (gelatine of animal origin) | 0.513 | 2x1 caps.  with meal | PL |
|  | C9 | 60 | 300 mg of freeze-dried juice from organic beetroot; micronized apple fibre; cellulose capsule shell | 0.286 | 2x1 caps. before meal | PL |
|  | C10 | 60 | 400 mg of beetroot extract including nitrates (40 mg); Vcaps cellulose capsule; hydroxypropyl methylcellulose; 70 mg of inulin Orafti GR | 0.393 | 1x1 caps. | PL |
|  | C11 | 100 | 200 mg of red beetroot extract; 200 mg of young barley extract (Hordeum vulgare); capsule (glazing agent: hydroxypropyl methylcellulose); 60 mg of niacin; 18 mg of pantothenic acid; bulking agent: microcrystalline cellulose; 4.2 mg of riboflavin; 4.2 mg of vitamin B6; 3.3 mg of thiamine; 600 µg of folic acid; 7.5 µg of vitamin B12 | 0.515 | 1x1 caps. | PL |
|  | C12 | 30 | 550 mg of beetroot extract; glazing agent: pullulan | 0.578 | 2x1 caps. | PL |
|  | C13 | 100 | 300 mg of 10:1 beetroot extract; capsule: vegetable cellulose | 0.401 | 2x1 caps. | PL |
|  | C14* | 60 | 400 mg of powdered beetroot; 40 mg of vitamin C; 10 mg of iron | 0.498 | 1x2 caps. | PL |
| powders | P1* | 60 | 1 serving (0.72 g) contains: 42 mg of iron fumarate 42 mg; including 14 mg of iron; 80 mg of vitamin C; 600 mg of powdered beetroot | 0.72 | 1 measure (0.72 g) | PL |
|  | P2 | 60 | powdered beetroot, whole ground | 15 | Mix 1 tablespoon of the product with water, juice or other drink. | PL |
|  | P3 | ND | powdered organic beetroot | 3.0 | Mix 1 teaspoon (3 g) of the product with 200 ml of water, juice, or add to other food. | IT |
|  | P4 | 66 | powdered red beetroot | 5.0 | Add 1-2 teaspoons of beetroot powder (5-10 g) to drinks or foods. | PL |
|  | P5 | 10-20 | powdered beetroot | 9.5 | 1x1 spoon | USA |
|  | P6 | 35 | beetroot tuber extract | 5.0 | 3x1 teaspoon | CZ |
|  | P7 | 25 | powdered beetroot | 3.0 | 1x1 flat teaspoon | EG |
|  | P8 | ND | powdered beetroot, whole ground | 15 | 1 spoon | HR |
|  | P9 | ND | powdered organic beetroot | 5.0 | 1x3 teaspoons | DE |
|  | P10 | ND | powdered beetroot | 1.0 | 1x half of flat teaspoon | PL |
|  | P11 | 100 | powdered organic beetroot | 3.0 | 3 flat teaspoons | CN |
|  | P12 | ND | powdered beetroot | 10 | 1 spoon | USA |
|  | P13 | 24 | crystals made of concentrated beetroot juice, including nitrates (4 g/100 g) | 5.5 | 2-3 measures, 1-3 hours before training or just after training | IE |

^1^ for some products the manufacturer did not provide the weight of the dosage unit, so it was determined according to the USP 43-NF 38 protocol <2091> Weight variation of dietary supplement; ^2^for all capsules, the average weight of the capsule contents was determined according to the USP 43-NF 38 protocol <2091> Weight variation of dietary supplement; ^3^for powders, the exact dose of the product was often not marked, so it was assumed that a spoon is 15 g, a teaspoon is 5 g, a flat teaspoon is 3 g, unless the manufacturer specified otherwise; ND – lack of data on the package

Table S2 The content of elements in beetroot-based DSs (x_m_ ± U, (k = 2)).

|  | Na | K | Ca | Mg | Fe | Mn | Zn | Cu | Mo | Cr | Co | Sr | Ba | Cd | Al | V |
| --- | --- | --- | --- | --- | --- | --- | --- | --- | --- | --- | --- | --- | --- | --- | --- | --- |
|  | µg/day ± U | µg/day  ± U | µg/day  ± U | µg/day  ± U | µg/day  ± U | µg/day  U | µg/day  U | µg/day  U | µg/day  U | µg/day  U | µg/day  U | µg/day  U | µg/day  U | µg/day  U | µg/day  U | µg/day  U |
| T1 | 2573 ±  48 | 2010.8 ±  3.7 | 49757 ±  68 | 729.2 ±  6.2 | 28.92 ±  0.13 | 16.009 ±  0.025 | 3.12 ±  0.14 | 3.215 ±  0.031 | 0.3416 ±  0.0091 | 0.2153  0.0035 | <LOQ | 8.092 ±  0.062 | 0.9713 ±  0.0067 | <LOQ | 5.172 ±  0.064 | <LOQ |
| T2 | 1260 ±  15 | 6185 ±  112 | 333.8 ±  3.7 | 1867.8 ±  7.9 | 11438 ±  105 | 41.49 ±  0.47 | 1.89 ±  0.14 | <LOQ | 21.27 ±  0.11 | 2.675  0.028 | <LOQ | 1.9152 ±  0.0103 | 1.6349 ±  0.0063 | <LOQ | 12.11 ±  0.11 | <LOQ |
| T3 | 2839 ±  18 | 12416 ±  152 | 909.5 ±  7.6 | 3422 ±  27 | 3633.4 ±  9.2 | 31.84 ±  0.21 | 10.34 ±  0.23 | <LOQ | 7.05 ±  0.11 | <LOQ | <LOQ | 6.17 ±  0.12 | 2.423 ±  0.019 | <LOQ | 8.15 ±  0.10 | <LOQ |
| T4 | 3198 ±  39 | 15313 ±  10 | 961 ±  13 | 2387.39 ±  6.05 | 7394 ±  45 | 37.51 ±  0.18 | 8.23 ±  0.33 | <LOQ | 13.81 ±  0.13 | <LOQ | <LOQ | 6.3531 ±  0.0195 | 2.477 ±  0.016 | 3.276 ±  0.082 | 9.26 ±  0.14 | <LOQ |
| T5 | 1812 ±  20 | 10385 ±  14 | 440.3 ±  0.93 | 2993 ±  25 | 100.56 ±  0.69 | 21.330 ±  0.066 | <LOQ | <LOQ | 1.553 ±  0.015 | 0.525  0.017 | <LOQ | 3.324 ±  0.051 | 2.745 ±  0.030 | <LOQ | 28.39 ±  0.25 | <LOQ |
| T6 | 2726 ±  9 | 2265 ±  10 | 50055 ±  181 | 703.9 ±  5.0 | 36.44 ±  0.20 | 16.681 ±  0.077 | 0.777 ±  0.091 | <LOQ | 0.3185 ±  0.0070 | 0.3658 | <LOQ | 5.816 ±  0.027 | 0.8068 ±  0.0035 | <LOQ | 6.195 ±  0.091 | <LOQ |
| T7 | 1312 ±  10 | 963.6 ±  3.2 | 27565 ±  181 | 335.90 ±  0.67 | 18.71 ±  0.17 | 8.671 ±  0.014 | <LOQ | <LOQ | 0.1103 ±  0.0046 | 0.1873 | <LOQ | 3.160 ±  0.026 | 0.3938 ±  0.0020 | <LOQ | 2.590 ±  0.063 | <LOQ |
| T8 | 1449 ±  26 | 22.3 ±  2.1 | 60.3 ±  2.2 | 342.5 ±  2.9 | 106.78 ±  0.79 | <LOQ  0.00 | <LOQ | <LOQ | 7.74 ±  0.14 | 4.479 | <LOQ | <LOQ | 3.088 ±  0.025 | 64.2  1.2 | 18.92 ±  0.10 | <LOQ |
| T9 | 3015 ±  16 | 8049 ±  202 | 309165 ±  920 | 1416 ±  11 | 93.63 ±  0.32 | 25.34 ±  0.21 | <LOQ | <LOQ | 1.250 ±  0.037 | <LOQ | 1.968 ±  0.089 | 47.99 ±  0.25 | 3.875 ±  0.025 | <LOQ | 27.28 ±  0.16 | <LOQ |
| T10 | 2851 ±  21 | 12022 ±  49 | 1426.2 ±  4.3 | 4350 ±  18 | 3752 ±  47 | 28.09 ±  0.18 | 10.82 ±  0.31 | <LOQ | 7.313 ±  0.076 | <LOQ | <LOQ | 7.586 ±  0.027 | 3.403 ±  0.011 | <LOQ | 17.71 ±  1.79 | <LOQ |
| C1 | 3478 ±  14 | 12868 ±  30 | 786.36 ±  3.95 | 1715 ±  12 | 113.1 ±  1.1 | 15.894 ±  0.064 | <LOQ | <LOQ | 22.41 ±  0.12 | <LOQ | <LOQ | 1.521 ±  0.025 | 3.027 ±  0.018 | <LOQ | 87.73 ±  0.70 | <LOQ |
| C2 | 4061 ±  37 | 18171 ±  206 | 395.1 ±  4.6 | 432.5 ±  1.1 | 34.84 ±  0.27 | 7.301 ±  0.043 | 7.94 ±  0.17 | <LOQ | 1.175 ±  0.030 | <LOQ | <LOQ | 0.6676 ±  0.0071 | 1.1888 ±  0.0071 | <LOQ | 5.20 ±  0.12 | <LOQ |
| C3 | 4541 ±  39 | 40338 ±  595 | 4890 ±  52 | 5395.6 ±  5.2 | 163.8 ±  1.6 | 52.49 ±  0.28 | 47.41 ±  0.81 | 6.50 ±  0.16 | 0.841 ±  0.027 | 1.932  0.016 | <LOQ | 17.092 ±  0.055 | 374.4 ±  2.3 | 3.84 ±  0.13 | 7.86 ±  0.45 | <LOQ |
| C4 | 1470 ±  17 | 10491 ±  47 | 267.19 ±  0.94 | 2023 ±  15 | 43.52 ±  0.30 | 27.17 ±  0.12 | 7.52 ±  0.24 | <LOQ | 0.296 ±  0.019 | <LOQ | <LOQ | 0.919 ±  0.015 | 2.369 ±  0.011 | 2.325 ±  0.045 | 7.86 ±  0.21 | <LOQ |
| C5 | 6336 ±  48 | 12012 ±  88 | 278.0 ±  1.5 | 829.2 ±  5.5 | 52.22 ±  0.16 | 10.907 ±  0.079 | 4.45 ±  0.19 | <LOQ | 0.210 ±  0.026 | <LOQ | <LOQ | 1.1884 ±  0.0058 | 1.787 ±  0.010 | <LOQ | 29.35 ±  0.33 | <LOQ |
| C6 | 1484 ±  16 | 9397 ±  18 | 276.6 ±  2.8 | 1144 ±  13 | 47.57 ±  0.15 | 27.333 ±  0.096 | 7.91 ±  0.34 | <LOQ | 0.317 ±  0.024 | <LOQ | <LOQ | 0.9561 ±  0.0094 | 2.496 ±  0.019 | <LOQ | 7.739 ±  0.090 | <LOQ |
| C7 | 15760 ±  158 | 8312 ±  63 | 103.9 ±  5.2 | 516.9 ±  3.4 | 30.92 ±  0.15 | 2.253 ±  0.016 | <LOQ | <LOQ | 0.188 ±  0.016 | <LOQ | <LOQ | 0.8750 ±  0.0061 | 8.160 ±  0.048 | <LOQ | 2.865 ±  0.045 | <LOQ |
| C8 | 1425.93 ±  5.03 | 7497 ±  56 | 680.1 ±  1.9 | 1209 ±  54 | 4141 ±  40 | 14.436 ±  0.033 | 4.33 ±  0.16 | <LOQ | 10.21 ±  0.12 | <LOQ | <LOQ | 4.514 ±  0.030 | 1.547 ±  0.010 | <LOQ | 8.74 ±  0.12 | <LOQ |
| C9 | 2361 ±  10 | 8804 ±  30 | 529.0 ±  4.8 | 959.8 ±  6.3 | 39.67 ±  0.30 | 25.168 ±  0.063 | 13.28 ±  0.14 | 1.613 ±  0.042 | 0.1962 ±  0.0074 | <LOQ | <LOQ | 0.6835 ±  0.0033 | 1.4718 ±  0.0057 | <LOQ | 3.705 ±  0.039 | <LOQ |
| C10 | 194.1 ±  3.9 | 640 ±  15 | 85.7 ±  1.1 | 151.19 ±  0.71 | 9.072 ±  0.022 | 0.2869 ±  0.0047 | <LOQ | <LOQ | 0.1112  0.0039 | <LOQ | <LOQ | 0.5612 ±  0.0067 | 0.3783 ±  0.0020 | <LOQ | 0.700 ±  0.043 | <LOQ |
| C11 | 1357.2 ± 2.4 | 1074 ±  18 | 1930.2 ±  6.7 | 387.2 ±  2.5 | 15.723 ±  0.067 | 0.7004 ±  0.0093 | <LOQ | <LOQ | 0.3270  0.0088 | <LOQ | <LOQ | 3.628 ±  0.021 | 0.7583 ±  0.0026 | <LOQ | 1.602 ±  0.052 | <LOQ |
| C12 | 14101 ±  170 | 7926 ±  25 | 166.6 ±  5.8 | 506.2 ±  3.8 | 26.96 ±  0.13 | 2.2282 ±  0.0058 | <LOQ | <LOQ | 0.046  0.014 | <LOQ | <LOQ | 0.757 ±  0.012 | 1.4161 ±  0.0067 | 78.31 ±  0.86 | 4.399 ±  0.015 | <LOQ |
| C13 | 13118 ±  217 | 11007 ±  140 | 845 ±  13 | 1720 ±  14 | 61.65 ±  0.26 | 17.04 ±  0.13 | 15.17 ±  0.26 ± | 5.007 ±  0.051 | 0.279  0.010 | <LOQ | <LOQ | 7.389 ±  0.031 | 50.0 ±  1.4 | <LOQ | 19.87 ±  0.16 | <LOQ |
| C14 | 4027 ±  16 | 9845 ±  126 | 1597.2 ±  8.8 | 1590 ±  13 | 24678 ±  126 | 102.289 ±  0.083 | 20.69 ±  0.41 ± | 1.434 ±  0.018 | 34.97  0.17 | <LOQ | 3.220 ±  0.059 | 7.042 ±  0.018 | 35.36 ±  0.34 | <LOQ | 2.59 ±  0.019 | <LOQ |
| P1 | 784.2 ±  1.4 | 4977 ±  63 | 397 ±  10 | 736.8 ±  6.3 | 12786 ±  272 | 43.30 ±  0.17 | 4.486 ±  0.086 ± | <LOQ | 21.3  1.1 | 0.3924  0.0094 | 2.786  0.079 | 3.008 ±  0.042 | 0.961 ±  0.018 | <LOQ | 2.58 ±  0.24 | <LOQ |
| P2 | 19710 ±  255 | 152775 ±  1590 | 17846 ±  117 | 31710 ±  165 | 995.6 ±  8.6 | 1057.8 ±  2.9 | 365.6 ±  2.9 ± | 36.45 ±  0.44 | 3.57  0.15 | <LOQ | <LOQ | 78.68 ±  0.86 | 1172 ±  20 | <LOQ | 128.0 ±  2.3 | <LOQ |
| P3 | 19710 ±  72 | 54576 ±  720 | 26772 ±  90 | 8124 ±  90 | 196.08 ±  0.84 | 46.41 ±  0.26 | 88.6 ±  1.6 ± | 20.04 ±  0.43 | 3.090  0.060 | <LOQ | <LOQ | 101.9 ±  1.4 | 1085 ±  23 | <LOQ | 17.3 ±  1.0 | <LOQ |
| P4 | 30840 ±  390 | 144810 ±  1020 | 18160 ±  130 | 18740 ±  150 | 541.7 ±  6.4 | 418.8 ±  1.1 | 233.1 ±  1.9 ± | 41.08 ±  0.57 | 2.73  0.29 | <LOQ | 35.25 ±  0.37 | 80.18 ±  0.63 | 211.1 ±  3.1 | <LOQ | 27.9 ±  1.3 | <LOQ |
| P5 | 21261 ±  314 | 117810 ±  371 | 33298 ±  209 | 14402 ±  171 | 449.1 ±  2.6 | 172.1 ±  1.1 | 134.0 ±  1.7 ± | 18.38 ±  0.29 | 2.731  0.048 | <LOQ | <LOQ | 79.90 ±  0.84 | 657 ±  13 | <LOQ | 43.30 ±  0.79 | <LOQ |
| P6 | 41490 ±  240 | 171630 ±  2055 | 48870 ±  285 | 19781 ±  119 | 741.4 ±  0.81 | 99.50 ±  0.45 | 224.7 ±  3.6 ± | 93.90 ±  0.27 | 8.74  0.14 | <LOQ | <LOQ | 212.0 ±  2.1 | 1331 ±  11 | <LOQ | 122.6 ±  2.0 | <LOQ |
| P7 | 32523 ±  579 | 46464 ±  213 | 4518 ±  14 | 7845 ±  72 | 1105 ±  14 | 76.64 ±  0.14 | 47.31 ±  0.72 ± | 13.87 ±  0.15 | 2.468  0.029 | 1.575  0.030 | <LOQ | 63.96 ±  0.57 | 166.5 ±  3.3 | <LOQ | 537.0 ±  3.3 | 1.224 ±  0.045 |
| P8 | 34095 ±  375 | 261675 ±  1065 | 32085 ±  270 | 28725 ±  150 | 2877 ±  30 | 258.8 ±  1.5 | 398.4 ±  4.5 ± | 85.10 ±  0.59 | 12.42  0.51 | 18.60  0.21 | <LOQ | 99.1 ±  1.1 | 545.0 ±  5.1 | <LOQ | 2123 ±  20 | <LOQ |
| P9 | 141060 ±  1290 | 237525 ±  1260 | 22620 ±  270 | 37005 ±  345 | 4923 ±  32 | 383.4 ±  4.2 | 237.5 ±  2.4 ± | 68.10 ±  0.96 | 10.43  0.15 | 8.48  0.26 | <LOQ | 319.5 ±  2.4 | 830 ±  23 | <LOQ | 2420 ±  23 | 4.73 ±  0.56 |
| P10 | 12433 ±  140 | 7009 ±  84 | 83.3 ±  1.7 | 418.0 ±  2.3 | 20.330 ±  0.091 | 1.898 ±  0.013 | <LOQ | <LOQ | 0.390  0.012 | <LOQ | <LOQ | 0.6475 ±  0.0050 | 1.1375 ±  0.0050 | <LOQ | 1.555 ±  0.054 | <LOQ |
| P11 | 189576 ±  108 | 122112 ±  1395 | 8525 ±  20 | 23391 ±  243 | 965.3 ±  5.8 | 149.04 ±  0.79 | 117.9 ±  2.3 ± | 31.77 ±  0.79 | 1.935  0.090 | <LOQ | <LOQ | 164.61 ±  0.99 | 128.70 ±  0.90 | <LOQ | 411.2 ±  5.9 | <LOQ |
| P12 | 49120 ±  110 | 140650 ±  590 | 9906 ±  86 | 17869 ±  61 | 572.8 ±  5.4 | 144.3 ±  7.1 | 91.2 ±  2.9 ± | 16.95 ±  0.40 | 2.850  0.058 | <LOQ | <LOQ | 113.18 ±  0.67 | 577 ±  11 | <LOQ | 145.8 ±  2.6 | <LOQ |
| P13 | 203874 ±  4934 | 403524 ±  2178 | 1721 ±  14 | 8910 ±  165 | 452.27 ±  2.97 | 341.02 ±  0.92 | 288.9 ±  2.1 ± | 10.69 ±  0.48 | 0.743  0.096 | <LOQ | 13.20 ±  0.30 | 5.651 ±  0.083 | 16.954 ±  0.083 | <LOQ | 141.5 ±  1.5 | <LOQ |

Table S3 Results of the realization of dietary recommendation (%) for selected elements according to Jarosz et al. [37] by a daily portion of DSs (EDI).

| Product | Na  AI  AI 1500 mg/day | K  AI  AI 3500 mg/day | Ca  RDAs 1000 mg/day | Mg  RDA man 420 mg/day | Fe  RDA men 10 mg/day | Mn  AIs man 2.3 mg/day | Zn  RDAs men 11 mg/day | Cu  RDA 0.9 mg/day |
| --- | --- | --- | --- | --- | --- | --- | --- | --- |
| T1 | 0.17 | 0.057 | 4.98 | 0.18 | 0.29 | 0.70 | 0.028 | 0.36 |
| T2 | 0.084 | 0.18 | 0.033 | 0.47 | 114 | 1.8 | 0.017 | NC |
| T3 | 0.19 | 0.35 | 0.091 | 0.86 | 36 | 1.4 | 0.094 | NC |
| T4 | 0.21 | 0.44 | 0.096 | 0.60 | 74 | 1.6 | 0.075 | NC |
| T5 | 0.12 | 0.297 | 0.044 | 0.75 | 1.01 | 0.93 | NC | NC |
| T6 | 0.18 | 0.065 | 5.01 | 0.18 | 0.36 | 0.73 | 0.0071 | NC |
| T7 | 0.087 | 0.028 | 2.8 | 0.084 | 0.19 | 0.38 | NC | NC |
| T8 | 0.10 | 0.00064 | 0.0060 | 0.086 | 1.07 | NC | NC | NC |
| T9 | 0.20 | 0.23 | 31 | 0.35 | 0.94 | 1.1 | NC | NC |
| T10 | 0.19 | 0.34 | 0.14 | 1.09 | 38 | 1.2 | 0.098 | NC |
| C1 | 0.23 | 0.37 | 0.079 | 0.43 | 1.1 | 0.69 | NC | NC |
| C2 | 0.27 | 0.52 | 0.0395 | 0.11 | 0.35 | 0.32 | 0.072 | NC |
| C3 | 0.30 | 1.2 | 0.49 | 1.35 | 1.6 | 2.3 | 0.43 | 0.72 |
| C4 | 0.10 | 0.2997 | 0.027 | 0.51 | 0.44 | 1.2 | 0.068 | NC |
| C5 | 0.42 | 0.34 | 0.028 | 0.21 | 0.52 | 0.47 | 0.040 | NC |
| C6 | 0.10 | 0.27 | 0.028 | 0.29 | 0.48 | 1.2 | 0.072 | NC |
| C7 | 1.1 | 0.24 | 0.0104 | 0.13 | 0.31 | 0.10 | NC | NC |
| C8 | 0.10 | 0.21 | 0.068 | 0.30 | 41 | 0.63 | 0.039 | NC |
| C9 | 0.16 | 0.25 | 0.053 | 0.24 | 0.40 | 1.1 | 0.12 | 0.18 |
| C10 | 0.013 | 0.018 | 0.009 | 0.04 | 0.091 | 0.012 | NC | NC |
| C11 | 0.09 | 0.031 | 0.19 | 0.10 | 0.16 | 0.030 | NC | NC |
| C12 | 0.94 | 0.23 | 0.017 | 0.13 | 0.27 | 0.10 | NC | NC |
| C13 | 0.87 | 0.31 | 0.085 | 0.43 | 0.62 | 0.74 | 0.14 | 0.56 |
| C14 | 0.27 | 0.28 | 0.16 | 0.40 | 247 | 4.4 | 0.19 | 0.16 |
| P1 | 0.052 | 0.14 | 0.0397 | 0.18 | 128 | 1.9 | 0.041 | NC |
| P2 | 1.3 | 4.4 | 1.8 | 7.9 | 10 | 46 | 3.3 | 4.05 |
| P3 | 1.3 | 1.6 | 2.7 | 2.03 | 1.96 | 2.0 | 0.81 | 2.2 |
| P4 | 2.1 | 4.1 | 1.8 | 4.7 | 5.4 | 18 | 2.1 | 4.6 |
| P5 | 1.4 | 3.4 | 3.3 | 3.6 | 4.5 | 7.5 | 1.2 | 2.04 |
| P6 | 2.8 | 4.9 | 4.9 | 4.9 | 7.4 | 4.3 | 2.04 | 10 |
| P7 | 2.2 | 1.3 | 0.45 | 2.0 | 11 | 3.3 | 0.43 | 1.5 |
| P8 | 2.3 | 7.5 | 3.2 | 7.2 | 29 | 11 | 3.6 | 9.5 |
| P9 | 9.4 | 6.8 | 2.3 | 9.3 | 49 | 17 | 2.2 | 7.6 |
| P10 | 0.83 | 0.20 | 0.0083 | 0.10 | 0.203 | 0.08 | NC | NC |
| P11 | 13 | 3.5 | 0.85 | 5.8 | 9.7 | 6.5 | 1.1 | 3.5 |
| P12 | 3.3 | 4.02 | 0.99 | 4.5 | 5.7 | 6.3 | 0.83 | 1.9 |
| P13 | 14 | 12 | 0.17 | 2.2 | 4.5 | 15 | 2.6 | 1.2 |

NC – realisation Carnot be calculated because content of chosen element was below LOQ

Table S4 Comparison of EDI of DSs with chosen toxicological parameters. Results are expressed as percentage of realization.

| Product | Cd PTMI 1.75 mg/70 kg/month | Cd  permissible contamination  1 mg/kg | Al  PTWI  140 mg/70 kg/week | Ba  RfD 14 mg/70 kg/day |
| --- | --- | --- | --- | --- |
| T1 | NC | NC | 0.026 | 0.0069 |
| T2 | NC | NC | 0.061 | 0.012 |
| T3 | NC | NC | 0.041 | 0.017 |
| T4 | 5.62 | 168 | 0.046 | 0.018 |
| T5 | NC | NC | 0.14 | 0.020 |
| T6 | NC | NC | 0.031 | 0.0058 |
| T7 | NC | NC | 0.013 | 0.0028 |
| T8 | 110 | 1466 | 0.095 | 0.022 |
| T9 | NC | NC | 0.14 | 0.028 |
| T10 | NC | NC | 0.089 | 0.024 |
| C1 | NC | NC | 0.44 | 0.022 |
| C2 | NC | NC | 0.026 | 0.0085 |
| C3 | 6.6 | 119 | 0.039 | 2.67 |
| C4 | 3.99 | 124 | 0.039 | 0.017 |
| C5 | NC | NC | 0.15 | 0.013 |
| C6 | NC | NC | 0.039 | 0.018 |
| C7 | NC | NC | 0.014 | 0.058 |
| C8 | NC | NC | 0.044 | 0.011 |
| C9 | NC | NC | 0.019 | 0.011 |
| C10 | NC | NC | 0.0035 | 0.0027 |
| C11 | NC | NC | 0.0080 | 0.0054 |
| C12 | 134 | 6774 | 0.022 | 0.010 |
| C13 | NC | NC | 0.099 | 0.36 |
| C14 | NC | NC | 0.013 | 0.25 |
| P1 | NC | NC | 0.013 | 0.0069 |
| P2 | NC | NC | 0.64 | 8.4 |
| P3 | NC | NC | 0.086 | 7.7 |
| P4 | NC | NC | 0.14 | 1.5 |
| P5 | NC | NC | 0.22 | 4.7 |
| P6 | NC | NC | 0.61 | 9.5 |
| P7 | NC | NC | 2.7 | 1.2 |
| P8 | NC | NC | 11 | 3.9 |
| P9 | NC | NC | 12 | 5.9 |
| P10 | NC | NC | 0.0078 | 0.0081 |
| P11 | NC | NC | 2.06 | 0.92 |
| P12 | NC | NC | 0.73 | 4.1 |
| P13 | NC | NC | 0.71 | 0.12 |

NC – realization cannot be calculated because content of chosen element was below LOQ; PTWI – a Provisional Tolerable Weekly Intake; PTMI – a Provisional Tolerable Monthly Intake; RfD – oral reference dose
